# Viral miRNAs Alter Host Cell miRNA Profiles and Modulate Innate Immune Responses

**DOI:** 10.3389/fimmu.2018.00433

**Published:** 2018-03-06

**Authors:** Afsar R. Naqvi, Jennifer Shango, Alexandra Seal, Deepak Shukla, Salvador Nares

**Affiliations:** ^1^Department of Periodontics-Mucosal Immunology Laboratory, College of Dentistry, University of Illinois at Chicago, Chicago, IL, United States; ^2^Department of Microbiology and Immunology, University of Illinois at Chicago, Chicago, IL, United States; ^3^Department of Ophthalmology and Visual Sciences, University of Illinois Medical Center, Chicago, IL, United States

**Keywords:** viral microRNA, herpesvirus, oral keratinocytes, macrophages, phagocytosis, cytokines

## Abstract

Prevalence of the members of herpesvirus family in oral inflammatory diseases is increasingly acknowledged suggesting their likely role as an etiological factor. However, the underlying mechanisms remain obscure. In our recent miRNA profiling of healthy and diseased human tooth pulps, elevated expression of human herpesvirus encoded viral microRNAs (v-miRs) were identified. Based on the fold induction and significance values, we selected three v-miRs namely miR-K12-3-3p [Kaposi sarcoma-associated virus (KSHV)], miR-H1 [herpes simplex virus 1 (HSV1)], and miR-UL-70-3p [human cytomegalovirus (HCMV)] to further examine their impact on host cellular functions. We examined their impact on cellular miRNA profiles of primary human oral keratinocytes (HOK). Our results show differential expression of several host miRNAs in v-miR-transfected HOK. High levels of v-miRs were detected in exosomes derived from v-miR transfected HOK as well as the KSHV-infected cell lines. We show that HOK-derived exosomes release their contents into macrophages (Mφ) and alter expression of endogenous miRNAs. Concurrent expression analysis of precursor (pre)-miRNA and mature miRNA suggest transcriptional or posttranscriptional impact of v-miRs on the cellular miRNAs. Employing bioinformatics, we predicted several pathways targeted by deregulated cellular miRNAs that include cytoskeletal organization, endocytosis, and cellular signaling. We validated three novel targets of miR-K12-3-3p and miR-H1 that are involved in endocytic and intracellular trafficking pathways. To evaluate the functional consequence of this regulation, we performed phagocytic uptake of labeled bacteria and noticed significant attenuation in miR-H1 and miR-K12-3-3p but not miR-UL70-3p transfected primary human Mφ. Multiple cytokine analysis of *E. coli* challenged Mφ revealed marked reduction of secreted cytokine levels with important roles in innate and adaptive immune responses suggesting a role of v-miRs in immune subversion. Our findings reveal that oral disease associated v-miRs can dysregulate functions of key host cells that shape oral mucosal immunity thus exacerbating disease severity and progression.

## Introduction

Viruses often infect the oral cavity and multiple studies indicate an association of one or more human herpesvirus (HHV) in oral inflammatory diseases (1, 2). For instance, Kaposi Sarcoma-Associated Virus (KSHV) and human cytomegalovirus (HCMV) sequences have been detected in endodontic periapical tooth abscesses, while high prevalence of HCMV, Epstein–Barr Virus (EBV) and herpes simplex virus (HSV1) were reported in patients with periodontal disease (3–5). Although a role of HHV is proposed in augmenting oral diseases (6), there is little information on how they contribute toward pathogenesis.

miRNAs are ~21 nt, single stranded, non-coding RNAs. The mature miRNA guides the protein machinery to cognate mRNAs that eventually affects target stability or translation (7, 8). miRNAs, by fine tuning the transcriptome, regulate a plethora of fundamental biological processes including cell differentiation, signaling, cell death, and pathogen response (7–10). Recently, viruses especially DNA viruses, have been reported to encode miRNAs (11–13). To date, more than 450 different miRNAs of viral origin (v-miRs) have been identified and the list continues to expand (miRbase 21^1^). It is now firmly established that both host and viral miRNAs play crucial roles in viral tropism, pathogenesis and latency (14–16). v-miRs can accomplish this by regulating both viral and host transcripts highlighting their significant potential to alter virus–host interactions. Several v-miRs target viral genes to which they are antisense (e.g., EBV miR-BART2 targeting of BALF5), while others control transcripts emanating from other genomic locations (e.g., HSV1 miR-H6 targeting ICP4) (17, 18). Given their significant capacity to regulate hundreds of host transcripts, viral miRNAs can influence and potentially regulate the host transcriptome. Indeed, global v-miR targetome profiling has revealed the set of host genes under direct influence of v-miRs. However, the impact of v-miRs on the host miRnome has not been examined. This is significant as a single miRNA can simultaneously fine tune expression of hundreds of transcripts. Examining the contribution of v-miRs on the host miRnome would provide a deeper understanding of their contribution in host–pathogen interactions.

In the oral cavity, keratinocytes are the primary sites of infection for various herpesviruses. Given the ability of v-miRs to enter target cells by exploiting the exosomal pathway (19–21), we hypothesize that viral miRNAs can reach and modulate myeloid inflammatory cell [particularly macrophage (Mφ) and dendritic cells (DC)] function including initiation of innate immune responses and activation of adaptive immune effector cells, *viz*., T and B cells. Earlier studies have focused on individual cell types to study immune responses. However, these functions are systemic and influenced by information gathered from other cells, exosomes being one of the major paracrine mediators (22, 23). Myeloid cells including Mφ patrol portals of pathogen entry and, therefore, have a critical role in host–virus interactions. HHV have evolved to escape immunosurveillance; and while the role of viral proteins is well documented, the contribution of v-miR demands extensive investigation. This is of particular importance as v-miR can be packaged into exosomes and delivered into various recipient cells including myeloid cells suggesting a paracrine nature of v-miRs. However, the functional significance of these observations is poorly studied.

Herpesviruses encode several miRNAs which can regulate expression of both viral and host transcripts. Indeed, several studies have demonstrated pathway specific functions of v-miRs signifying their crucial role in viral tropism and lifecycle. However, the impact of v-miRs on the expression of non-coding RNAs has not been previously examined. Recently, our group for the first time demonstrated the presence and elevated expression of four HHV derived v-miRs in inflamed tooth pulps (24). We, therefore, investigated the global impact of oral inflammation associated v-miRs, *viz*., miR-K12-3-3p (KSHV), miR-H1 (HSV1), miR-UL70-3p (HCMV) on the expression of cellular miRNA. In this study, we demonstrate that disease associated HHSV miRNAs can alter cellular miRNA profiles in human oral keratinocytes (HOK), are secreted in high amounts in exosomes, and impact cell specific miRNA in Mφ. v-miR deregulated cellular miRs target endocytic, cytoskeletal organization related pathways and we validated three novel gene targets of the v-miRs related to these pathways. Functional analysis showed that miR-H1 and miR-K12-3-3p modulate phagocytosis and cytokine secretion in myeloid cells. These results provide a novel understanding on the role of v-miRs in modulating host cellular functions, subverting immune responses and their likely role in the pathogenesis of oral inflammatory diseases.

## Materials and Methods

### Primary Human Monocyte Isolation and Macrophage Differentiation

Monocytes were isolated from freshly prepared buffy coats collected from healthy donors (*n* ≥ 3, Sylvan N. Goldman Oklahoma Blood Institute, Oklahoma City, OK, USA) by density gradient centrifugation and magnetic bead sorting as previously described (25–28).

### Primary HOK Isolation and Culture

Primary HOK were purchased from ScienCell Research Laboratories (Carlsbad, CA, USA). HOK are negative for HIV-1, HBV, HCV, mycoplasma, bacteria, yeast, and fungi. Cells were cultured using Keratinocyte Medium (LifeLine Cell Technology, Friedrich, MD) which contains basal medium, growth supplements (hormones, growth factors, and proteins) and penicillin/streptomycin.

In addition to primary HOK, we also used immortalized human gingival epithelial cells (kindly provided by Dr. Richard Lamont, University of Louisville, KY, USA). These cells were cultured in flasks containing keratinocyte growth medium [Keratinocytes Basal Media 2 plus growth supplements (Lonza Group Ltd., Basel, Switzerland) containing 0.1 mM calcium].

### Detection of Virus in Primary HOK and Macrophages

To rule out any possible contamination of HCMV and HSV1 viruses in our primary cell cultures, we tested for viral genomic DNA. Total DNA isolated from HOK and Mφ was screened for viral genome by PCR-based method. Positive and negative controls were included. We used HSV1 and HCMV detection kits from Norgen Biotek Corp. (ON, Canada). Mφ and HOK genomic DNA was isolated per manufacturers’ instruction. PCR was performed using virus-specific primers provided in the kit and the products were resolved on 2% agarose gel. As a positive control, isolation control and a PCR control were included.

### Macrophage and Oral Epithelial Cell Transient miRNA Transfections

Transient transfections were performed using Lipofectaine 2000 reagent (Life Technologies, San Diego, CA, USA) according to manufacturer’s instructions. Red siGLO oligos (Thermo Fisher Scientific, Waltham, MA, USA) were used to determine transfection efficiency. After 36 h, cells were harvested for protein detection or RNA isolation. Cells were transfected with viral miRNA mimics (Qiagen, Gaithsburg, MD, USA) at a final concentration of 15 nM for 36 h. Controls consisted of mock transfected and control mimic (Qiagen) transfected cultures. Levels of viral miRNAs were used to determine transfection efficiency using miScript PCR primer assays as described above.

### Cell Viability Assay

Cell viability was determined using the CellTiter 96 AQueous Cell Proliferation Assay Kit (Promega, Madison, WI, USA) according to manufacturer’s instructions. A total of 20 µL of MTS (3-(4,5-dimethylthiazol-2-yl)-5-(3-carboxymethoxyphenyl)-2-(4-sulfophenyl)-2H-tetrazolium) reagent was added to each well and incubated for 2 h. Absorbance at 490 nm was monitored using the SpectraMax^®^ M2 (Molecular Devices, Sunnyvale, CA, USA) plate reader.

### miRNA Profiling Using Exiqon Microarrays

v-miRNA transfected cells were harvested after 40 h, and total RNA was isolated using the miRNeasy kit (Qiagen). RNA integrity was assessed using the Nanodrop (Thermo Fisher Scientific) and 2100 Bioanalyzer (Agilent, Foster City, CA, USA). miRNA expression was performed by Exiqon Services (Vedbaek, Denmark) by use of seventh-generation microarrays (miRBase v.19). Total RNA (225 ng) was labeled using the miRCURY LNA microRNA Hi-Power Labeling Kit Hy3/Hy5 and subsequently hybridized onto miRCURY LNA microRNA arrays, following the procedures described by the manufacturer. Data normalization were performed by Exiqon using Quantile normalization. Initial analysis was performed by Exiqon using R/bioconductor, primarily by use of the limma package (Exiqon). Expression analysis of variance over time was performed with *p*-values adjusted using the Benjamini–Hochberg method and identified genes subjected to the Tukey’s “honestly significant difference” test (29). We followed the MIAMI protocol and deposited the data in the Omnibus repository with the accession number GSE107674.

### Isolation of Exosome from KSHV-Infected Primary Effusion Lymphoma (PEL) Cell Line BC-3

Cell lines were cultured in RPMI1640 media (Fisher Scientific, Hampton, NH, USA) containing a final concentration of 2 mM l-glutamine, 10% exosome-depleted fetal bovine serum (Thermo Fisher Scientific), penicillin G (100 U/mL), and streptomycin sulfate (100 µg/mL), at 37°C in 5% CO_2_. PEL cell lines BC-3, as well as Burkitt’s lymphoma cell line BJAB, were grown to a cell density of 0.05 to 0.5 million cells/mL, spun at 300 × *g* for 5 min, and resuspended in exosome-depleted RPMI 1640 media at day 0. Cell cultures (25 mL) were harvested at days 3 and 6 by centrifuging for 300 × *g* for 5 min to separate cells and supernatant fractions. Cell pellets were resuspended at a maximum of approximately 10 million cells per mL of Qiazol (Qiagen), and total RNA was isolated.

### Total RNA Isolation and Quantitative Real-time PCR

Cells were lysed and total RNA (including miRNAs) isolated using the miRNeasy kit (Qiagen) according to manufacturer’s instructions. For mature v-miR or cellular miRNA quantification, miScript primers and miScript II RT Kit were purchased from Qiagen. 100 ng total RNA was reverse transcribed according to manufacturer’s instruction. The reactions were run using miRNA specific primer and universal primer (both from Qiagen) in the PCR mix buffer containing SYBR Green (Roche, Indianapolis, IN, USA). RNU6B was used as endogenous control. The Cq values of replicates were analyzed to calculate relative fold change using the delta-delta Cq method and the normalized values plotted as histograms with SD.

### Flow Cytometry

Cells were harvested after treatments, washed with ice cold PBS, and fixed with 2% paraformaldehyde (PFA) for 15 mins. After washing, cells were resuspended in 50–100 µL of FcR blocking reagent (Miltenyi Biotech, Bergisch Gladbach, Germany), followed by 15 min incubation at room temperature (RT) to allow blocking of Fc receptors. The cell pellet was washed twice, resuspended in 50 µL 2% BSA/TBS (w/v), and incubated with fluorochrome-conjugated antibodies. Samples were analyzed using a FACScan or BD Cyan flow cytometer using CellQuest software (BD Biosciences, San Jose, CA, USA). Further analysis was performed using FlowJo software (Tree Star Inc., Ashland, OR, USA).

### Exosome Isolation

Conditioned media were collected from cultured cells (HOK, BC-3 or Mφ), centrifuged at 17,000 × *g* for 1 h, and supernatant were carefully collected. Exosomes were isolated using ExoQuick (System Biosciences, Mountain View, CA, USA) according to manufacturer’s instruction. Briefly, 10 mL of supernatant was incubated with 2 mL of ExoQuick overnight at 4°C then centrifuged at 1,600 × *g* for 30 min. Supernatant was removed carefully and the pellet was resuspended in ~50–100 μL PBS.

### Electron Microscopy

Exosomes stained with the vesicle pellet were resuspended in 4% PFA and deposited onto formvar/carboncoated EM grids. The vesicle-coated grids were washed twice with PBS (3 min each), twice with PBS/50 mM glycine, and finally with PBS/0.5% BSA (10 min); stained with 2% uranyl acetate; and then viewed for transmission EM using a Zeiss EM900 (Carl Zeiss, Oberkochen, Germany).

### Exosome Size Determination

The concentration and size distribution of the isolated exosomes were measured using NanoSight Tracking Analysis (NTA) (Salisbury, United Kingdom). Prior to sampling, the sample solutions were homogenized by vortexing, followed by final dilution of 1:100 to 1:200 in 0.2-μm filtered 1× PBS. Prior to running the samples, traceable 97 ± 3 nm polystyrene latex standards were analyzed using the Malvern NanoSight LM10-HS (Northwestern University, Evanston, IL, USA). Subsequently, diluted exosome samples were run and images and videos captured. A blank 0.2-μm filtered 1× PBS was also run as a negative control. Each sample analysis was conducted for 90 s. The Nanosight automatic analysis settings [high sensitivity, blue laser (405 nm, 645 mW)] were used to process the data. All samples were evaluated in triplicate.

### v-miR Transfection of Exosomes

Exosomes isolated from HOK were transfected using Exo-fect (System Biosciences, Mountain View, CA, USA) as per manufacturer’s instructions. Briefly, 100 µg exosomes were transfected with 20 nM of v-miR or control mimics using 10 µL Exo-Fect and incubated for 37°C for 10 min. Reaction was stopped by adding ExoQuick and exosomes were pelleted after 30 min by centrifuging at 17,000 × *g*/3 min. Exosomes were resuspended in 500 µL serum-free media and incubated with the recipient cells at two different concentrations (100 and 250 µL) overnight before performing functional assays.

### Western Blot

Cells transfected with v-miRNA mimics, or control mimics were harvested and lysed in cell lysis buffer (Cell Signaling Technology, Danvers, MA, USA) supplemented with protease inhibitors (Roche, Basel, Switzerland). Lysates were incubated on ice for 30 min and were clarified using centrifugation at 17,000 × *g* for 15 min at 4°C, and protein content was estimated using the Bradford assay (Bio-Rad Laboratories, Hercules, CA, USA). Exosomes were directly lysed in sample buffer. Protein detection was performed as described earlier.

### Exosome-Binding Assays

Human oral keratinocytes exosomes were labeled with RNA binding red fluorescent dye while, exosome protein was labeled with green fluorescent dye using Exo-Glow labeling kit (SBI, Mountain View, CA, USA) according to the supplier’s protocol. Day 7, differentiated Mφ were incubated with fluorescently labeled exosomes (10 µg protein/10^6^ cells) for 16 h. Cells were washed thrice with PBS, treated with Accutase (STEMCELL Technologies Inc., Cambridge, MA, USA) for 5 min to remove surface adhered, non-internalized exosomes. Mφ were fixed with 2% PFA, mounted with Prolong Gold antifade (ThermoFisher Scientific) and imaged on Zeiss LSM 710 confocal microscope. Uptake of exosomes was also examined by real-time PCR using v-miR specific primers. As a negative control, prior to incubation with exosomes, cells were treated with cytochalasin D (5 µg/mL, Sigma-Aldrich, St. Louis, MO, USA) to block exosome uptake by inhibiting actin polymerization.

### Pathway Prediction Analysis

For each sample set, all the altered miRNAs were uploaded onto DIANA-miRPath^2^ for pathway prediction. Because not all the miRNA targets have been validated, we selected DIANA-microT-CDS algorithm which included predicted miRNA targets.

### *In Silico* Viral miRNA Target Predictions and Seed Match Analyses

To identify the viral miRNA targets, we used viR-miR database.^3^ Viral miRNA sequences were entered into the search database and list of genes were procured as an output file. As an alternate, we also searched the 3′UTR of select genes employing the RNA hybrid algorithm that is also used by viR-miR database. Genes with known functions in endocytosis and cellular trafficking were selected for further studies.

### Luciferase Reporter Constructs and Dual Luciferase Reporter Assays

Genomic DNA were isolated from freshly prepared PBMCs using QIAamp DNA mini kit (Qiagen) according to manufacturer’s instructions. The 3′UTRs of predicted miRNA target genes were PCR amplified using Phusion Taq polymerase (NEB, Ipswich, MA, USA). The amplified products were digested with restriction enzymes (Xho I and Not I) and ligated downstream to the luciferase reporter gene in psiCHECK™-2 vector (Promega). Dual luciferase assays were performed as described earlier (26, 28, 30).

### Phagocytosis Assay and Imaging

Mφ cultured at a density of 400,000/well (96-well plate) were transfected on day 7 with miScript viral miRNA or control miRNA mimics (Qiagen). After 24 h, phagocytosis assay was performed with pHrodo Red *Escherichia coli* bioparticles conjugate (Invitrogen), as previously described (26). Briefly, the labeled bioparticles were resuspended in Live Imaging Buffer (Life Technologies) and homogenized by sonication for 2 min. Culture media was replaced with resuspended labeled *E. coli* and incubated for 1 h.

Similar experiments were performed with U937 differentiated macrophages. HOK-derived exosomes were transfected with miR-K12-3-3p or control mimic as described above. After 24 h, cells were assayed for *E. coli* phagocytosis and incubated for 4 h. As a negative control, cells were treated with 5 mM cytochalasin D (Sigma-Aldrich) prior to adding bioparticles. The cells were washed three times with PBS, fixed with 4% PFA, and analyzed by flow cytometry. Images were captured using a Zeiss LSM 710 confocal microscope (Carl Zeiss, Oberkochen, Germany) with 403/1.2 Water DIC C-Apochromat objective and 2× zoom or EVOS florescent microscope (Life Technologies) at original magnification. Confocal images were processed on ZEN lite software. Images were captured for four independent, randomly selected fields for each donor.

### Cytokine Analysis

Supernatants were collected from Mφ challenged with *E. coli* for 4- and 18-h and analyzed for cytokine/chemokine levels by ELISA or multiplex assays. Multiplex analysis of 23 different secreted cytokines/chemokines was performed using Milliplex (Millipore, Billerica, MA, USA). Data were collected on Bio-Plex flow cytometer (Bio-Rad, Hercules, CA, USA).

### Statistical Analysis

Data were analyzed on GraphPad Prism (LaJolla, CA, USA). The results are represented as mean ± SEM of 3–5 independent replicates and experiments were conducted at least thrice. *p*-Values were calculated using Student’s *t*-test, and *p* < 0.05 were considered significant.

## Results

### Enforced Expression of v-miRs Alters Cellular miRNA Profiles in HOK

Since oral keratinocytes are the primary sites of infection for various HHV, we questioned whether induced expression of v-miRs observed in clinical samples of pulpitis (24) and periodontitis (Naqvi et al., submitted) can impact cellular miRNA profiles. To test this hypothesis, we selected three different v-miRs identified in our previous studies namely miR-K12-3-3p, miR-H1, and miR-UL-70-3p that belong to Kaposi sarcoma-associated virus (KSHV), herpes simplex virus 1 (HSV1), and HCMV, respectively [(24); Naqvi et al., submitted]. Primary HOK cultures were first screened for the presence of HSV1 and HCMV DNA by PCR. No viral genome contamination was detected in any of the donor cultures utilized (Figure S1 in Supplementary Material). Using a DY-547 dye labeled siRNA (siGLO) we obtained high transfection efficiency (>90%) in HOK (Naqvi et al., submitted). Further, to examine the impact of v-miR mimics on HOK viability, cells were transfected with 15 nM of v-miR mimics and cell viability was assessed after 36 h. We noted that v-miR or control mimic transfected cells show similar viability indicating that v-miRs does not induce apoptosis in cells within the time examined (Naqvi et al., submitted and Figure S2 in Supplementary Material).

**Figure 1:**
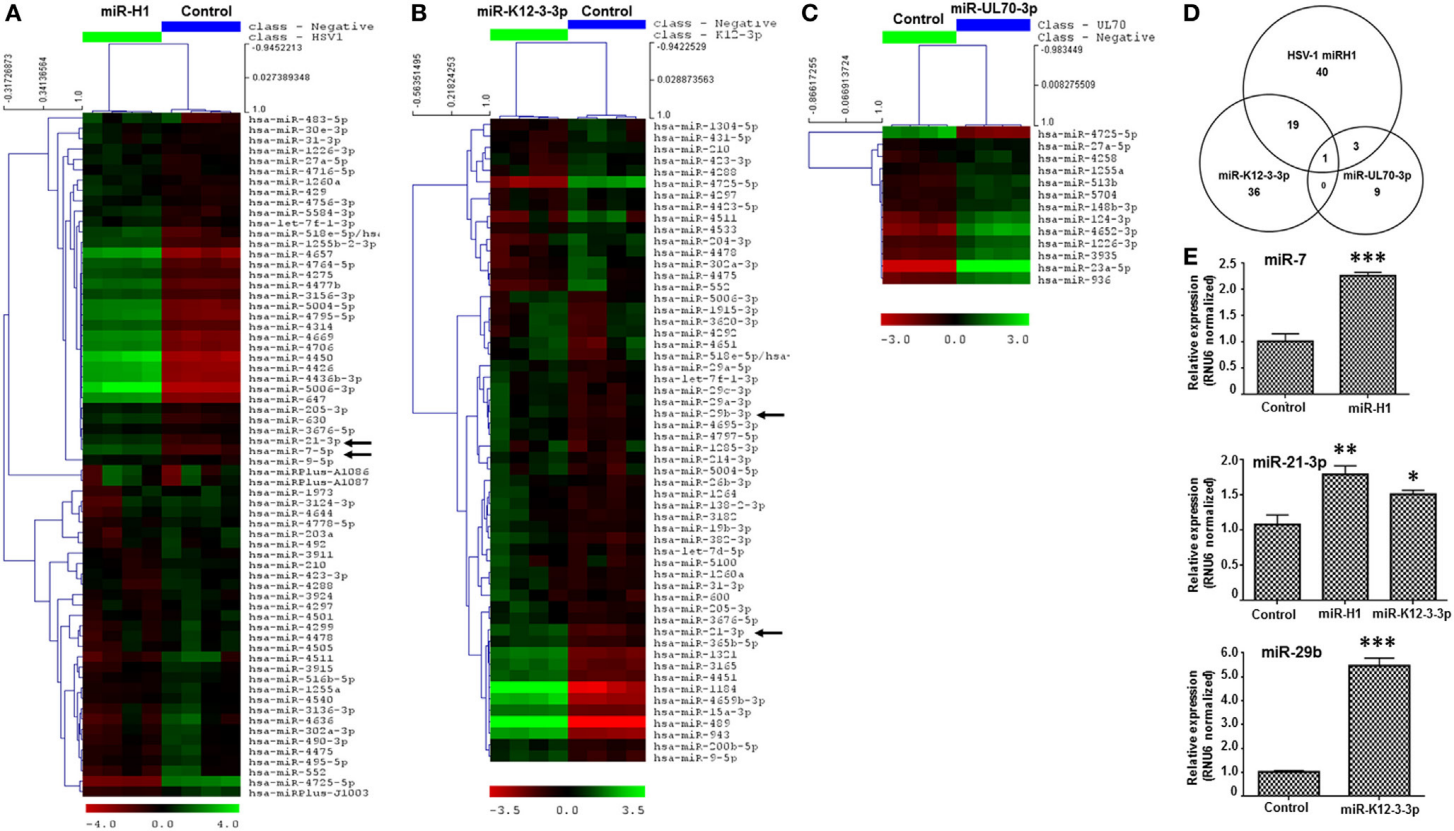
v-miRs modulate expression of cellular miRNAs in human oral keratinocytes (HOK). Heat maps showing differentially expressed cellular miRNAs in **(A)** miR-H1, **(B)** miR-K12-3-3p, and **(C)** miR-UL70-3p transfected HOK. **(D)** Venn diagram showing the distribution of unique and overlapping altered cellular miRNAs in v-miR-transfected HOK. **(E)** Validation of microarray by quantitative RT-PCR for miR-7, miR-21-3p, and miR-29b (marked by black arrows in the heat map) in another separate cohort of transfected HOK. RNU6 was used as endogenous control. Data are expressed as mean ± SEM of four independent transfections. Student’s *t*-test was conducted to calculate *p*-values (*p < 0.05, **p < 0.01, ***p < 0.001).

Microarray analysis of v-miRs transfected HOK revealed altered expression of several cellular miRNAs indicating that miR-H1 and miR-K12-3-3p but not miR-UL70-3p have a marked impact on the host miRnome compared with control miRNA mimic. Figure 1A shows a heat map of differentially expressed cellular miRNAs in miR-H1 transfected HOK. Compared to control mimic, expression of 63 miRNAs was significantly altered in miR-H1 overexpressing cells. Among these, expression of 33 was upregulated and 30 was downregulated. For miR-K12-3-3p expressing HOK, 56 differentially expressed miRNAs were identified, of which 41 were upregulated and 15 were downregulated (Figure 1B). Among the three v-miRs tested, UL-70-3p exhibited the least impact on target cell miRnome. We observed differential expression of only 13 miRNAs with 12 showing induced expression while, only 1 miRNA was downregulated (Figure 1C). The complete list of the differentially expressed miRNAs with fold change is provided in Tables S1–S3 in Supplementary Material. We also examined the common and unique set of miRNAs altered by v-miRs. Interestingly, we found only one miRNA that was common between miR-H1, miR-K12-3-3p, and miR-UL70-3p altered host miRNAs (Figure 1D). Between miR-H1 and miR-K12-3-3p, expression of 20 miRNAs was common while 43 and 36 unique miRNAs were identified in each dataset, respectively. Likewise, four miRNAs were shared to miR-H1 and miR-UL70-3p, while unique miRNAs in both datasets were 59 and 9, respectively. miR-K12-3-3p and miR-UL70-3p showed only one common miRNA. Overall, these results clearly show the specific impact of v-miRs on the host miRnome.

We selected three differentially expressed miRNAs, miR-7, miR-21-3p, and miR-29b, and checked their expression by quantitative RT-PCR in three independent donors. We noticed significant increase in the expression in all three tested miRNAs which corroborates with the microarray data (Figure 1E).

### v-miRs from HOK Exosomes Are Delivered to Recipient Macrophages

Exosomes are ~40–100 nm vesicles secreted by various cells. These extracellular vesicles, through endocytic pathways, can merge and deliver their contents, including proteins, mRNA, and miRNAs, into recipient cells (22, 23). Viral miRNAs, similar to host miRNAs, can be targeted to exosomal pathways through which they gain access to other cells (19–21). Upon endocytosis, the exosomes release their contents to recipient cells thereby potentially modulating their functions or responses. Indeed, we and others have shown that viruses exploit this pathway to dysregulate the information exchange (31–34). We thus hypothesize that v-miR can exploit this pathway to modulate immune cells in close proximity including Mφ, key tissue resident innate immune cells that respond to and trigger production of mediators of inflammation leading to the activation of the adaptive immune response.

**Figure 2:**
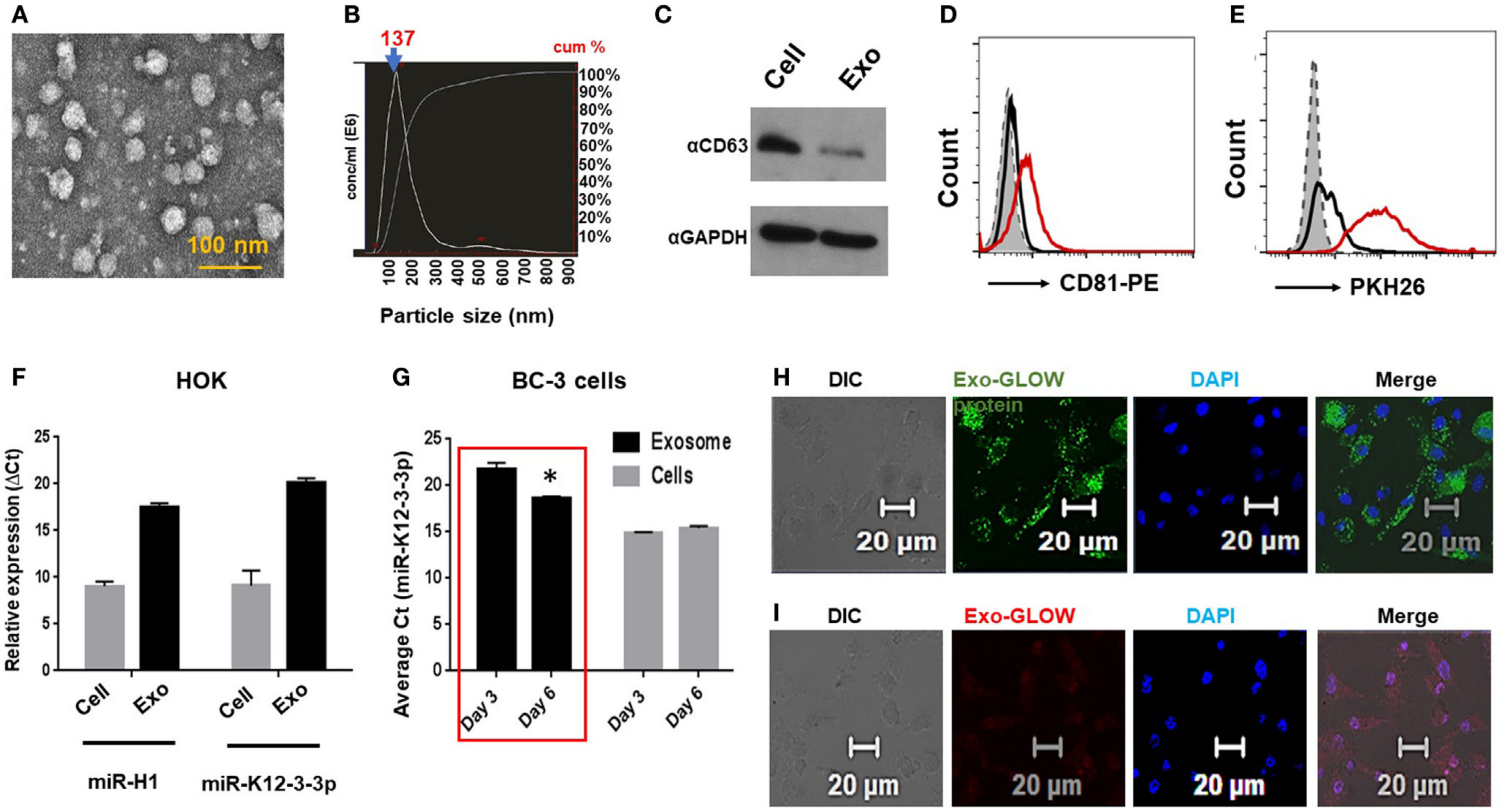
Oral keratinocytes derived exosomes carry v-miRs and deliver their contents into primary macrophages. **(A)** Electron microscopy image of exosomes isolated from human oral keratinocytes (HOK). Scale bar, 100 nm. **(B)** Size of the purified exosomes was analyzed on Nanosight Tracking Analysis. The particle size distribution and calculated original concentration of the HOK-derived exosomes. The arrow indicates the particle size (mode) of 137 nm. **(C)** Western blot showing detection of exosomal marker CD63 in the exosomal and cell lysates. Exosomes were bound to latex beads and then stained with exosome marker CD81 and labeled with lipid binding dye PKH26. Histograms showing **(D)** CD81 positive beads and **(E)** PKH26 staining indicating that isolated exosomes are membrane limiting vesicles. **(F)** Quantitative RT-PCR showing expression of v-miRs in cellular and exosomal RNA of miR-H1 transfected HOK. **(G)** High levels of miR-K12-3-3p secreted in the exosomes KSHV-infected BC-3 cells. miR-K12-3-3p expression in KSHV-infected cells and exosomes was analyzed by qPCR. Confocal images showing purified HOK exosomes deliver **(H)** protein and **(I)** RNA contents inside primary human Mφ. Scale bar, 20 µm. Student’s *t*-test was conducted to calculate *p*-values (*p < 0.05).

To investigate this possibility, exosomes were first isolated from primary HOK and preparations characterized by electron microscopy (Figure 2A). We also determined exosome size by NTA and observed particle size of 130 nm (Figure 2B, arrow). Further, we confirmed detection of the exosome marker CD63 in exosomal lysates by western blot and CD81 by flow cytometry analysis (Figures 2C,D). Finally, we show that membrane permeable dye PKH26 stains our exosome preparation suggesting intact membrane-bound vesicles (Figure 2E).

To show the release of v-miRs in exosomes, we examined their levels in v-miR transfected as well as latent virus infected cells. Using RT-qPCR analysis, we confirmed that miR-H1 and miR-K12-3-3p were packaged within exosomes derived from v-miR transfected HOKs (Figure 2F). Further, we checked miR-K12-3-3p levels in the KSHV-infected PEL cell line BC-3 at two different time points (3 and 6 day). High levels of miR-K12-3-3p were detected in both cells and exosomes. Importantly, we noted higher levels of exosomal miR-K12-3-3p postinfection that increased with time (Figure 2G; compare day 3 vs day 6). Together, these results show that v-miRs are packaged in exosomes derived from both v-miR transfected and virus-infected cells.

Next, we tested whether HOK-derived exosomes can deliver their contents to Mφ. To examine this aspect, HOK exosomes were labeled with RNA and protein dye and incubated with primary Mφ. We noticed efficient delivery of both RNA (red) and protein (green) into recipient Mφ (Figures 2H,I). Exosomes isolated from miR-K12-3-3p transfected HOK were incubated with Mφ overnight and the presence of v-miR was examined in the recipient cells by quantitative PCR. miR-K12-3-3p were detected (Cq ~27–29) in Mφ incubated with HOK exosomes (data not shown). Therefore, it is plausible that exosomal delivery of v-miRs to nearby immune cells (for instance Mφ) may negatively impact host defenses.

### miRNA Profiling Identifies Differentially Expressed Cellular miRNAs in v-miR-Transfected Primary Human Macrophages

**Figure 3:**
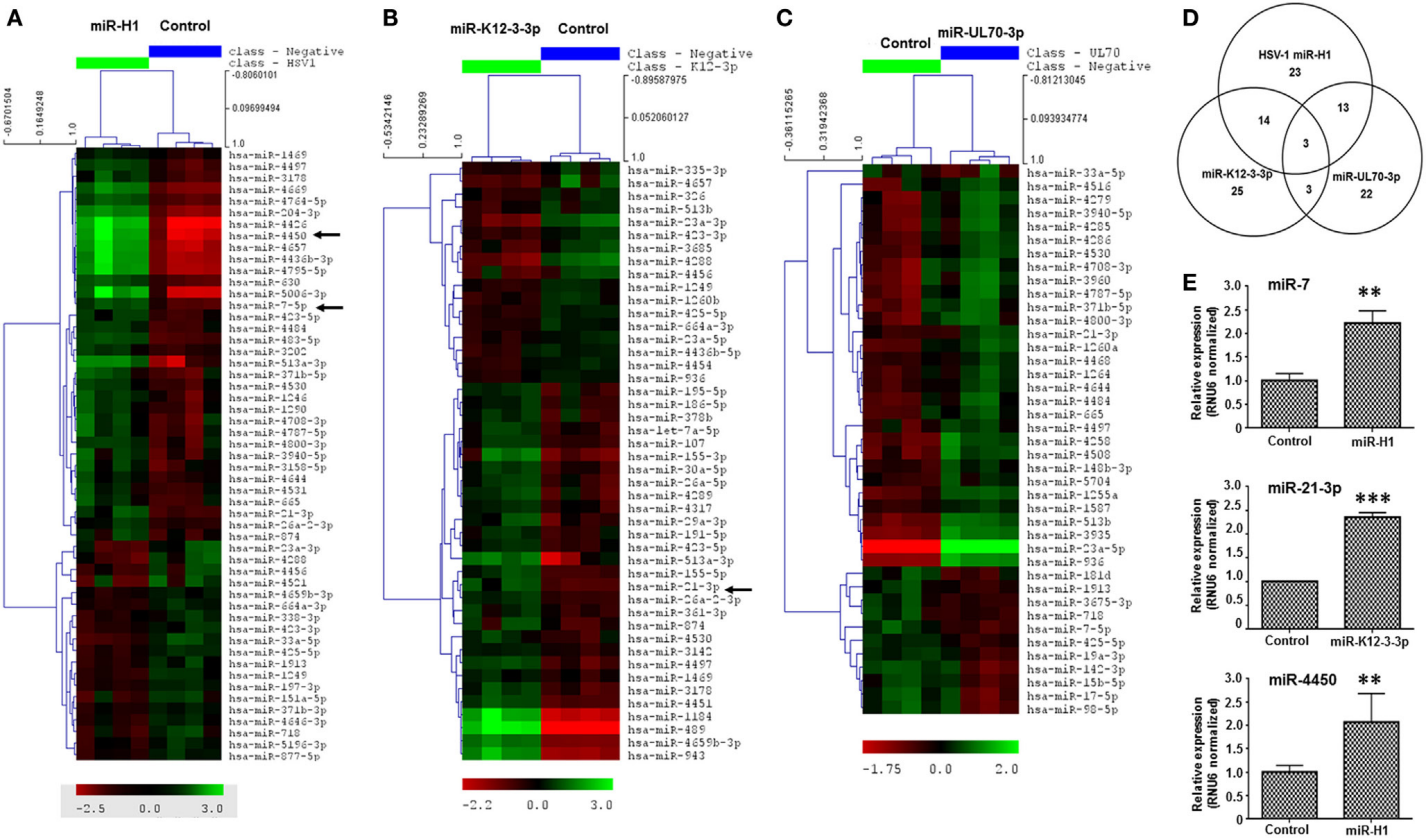
v-miRs modulate expression of cellular miRNAs in macrophages. Heat maps showing differentially expressed cellular miRNAs in **(A)** miR-H1, **(B)** miR-K12-3-3p, and **(C)** miR-UL70-3p transfected Mφ. **(D)** Venn diagram showing the distribution of unique and overlapping altered cellular miRNAs. **(E)** RT-qPCR validation of differentially expressed miRNAs miR-7, miR-21-3p, and miR-4450 identified in microarray data in a separate cohort of primary Mφ. RNU6 was used as endogenous control. Data are expressed as mean ± SEM of four independent transfections. Student’s *t*-test was conducted to calculate *p*-values (**p < 0.01, ***p < 0.001).

Having established that v-miRs are packaged within exosomes and are released inside recipient Mφ, we next examined the impact of these v-miRs on cellular miRNA profiles. v-miR transfected cells exhibited significant changes in the expression of various miRNAs in macrophages. Heat maps show the scaled expression of differentially expressed miRNA in miR-H1, K12-3-3p, and miR-UL70-3p transfected Mφ (Figures 3A–C). Altered expression of 53 miRNAs was observed in miR-H1 transfected Mφ of which 34 and 19 were upregulated and downregulated, respectively. We observed 55 differentially expressed miRNAs in miR-K12-3-3p expressing Mφ and among these 28 and 17 were upregulated and downregulated. For miR-UL70-3p, 41 (29 upregulated and 12 downregulated) miRNAs showed significant expression change in Mφ. Tables S4–S6 in Supplementary Material lists the fold change and *p*-values of differentially expressed miRNAs in Mφ.

We next evaluated common and unique sets of miRNAs impacted by the v-miRs. As shown in the Venn diagram (Figure 3D), three miRNAs were common to miR-H1, K12-3-3p, and miR-UL70-3p, while 17 were shared between miR-H1 and K12-3-3p, 16 were common to miR-H1 and miR-UL70-3p. Finally, six were shared between K12-3-3p and miR-UL70-3p. To identify cell type specific modulation of cellular miRNAs, we searched for miRNAs commonly altered by v-miRs in HOK and Mφ. We found 15, 9, and 8 miRNAs that were differentially altered in both cell types transfected with miR-H1, miR-K12-3-3p, and miR-UL70-3p, respectively (Figures S3A–C in Supplementary Material). Together, these results strongly validate our previous observation of v-miR specific changes in cellular miRNA repertoire and also suggest cell type restricted changes.

Microarray results were further validated by RT-qPCR analysis of three differentially expressed miRNAs, *viz*., miR-7, miR-21-3p, and miR-4450 (Figure 3E), in an independent cohort of four donors.

### v-miR-Mediated Changes in Cellular miRNAs Occurs at the Transcriptional or Posttranscriptional Level

**Figure 4:**
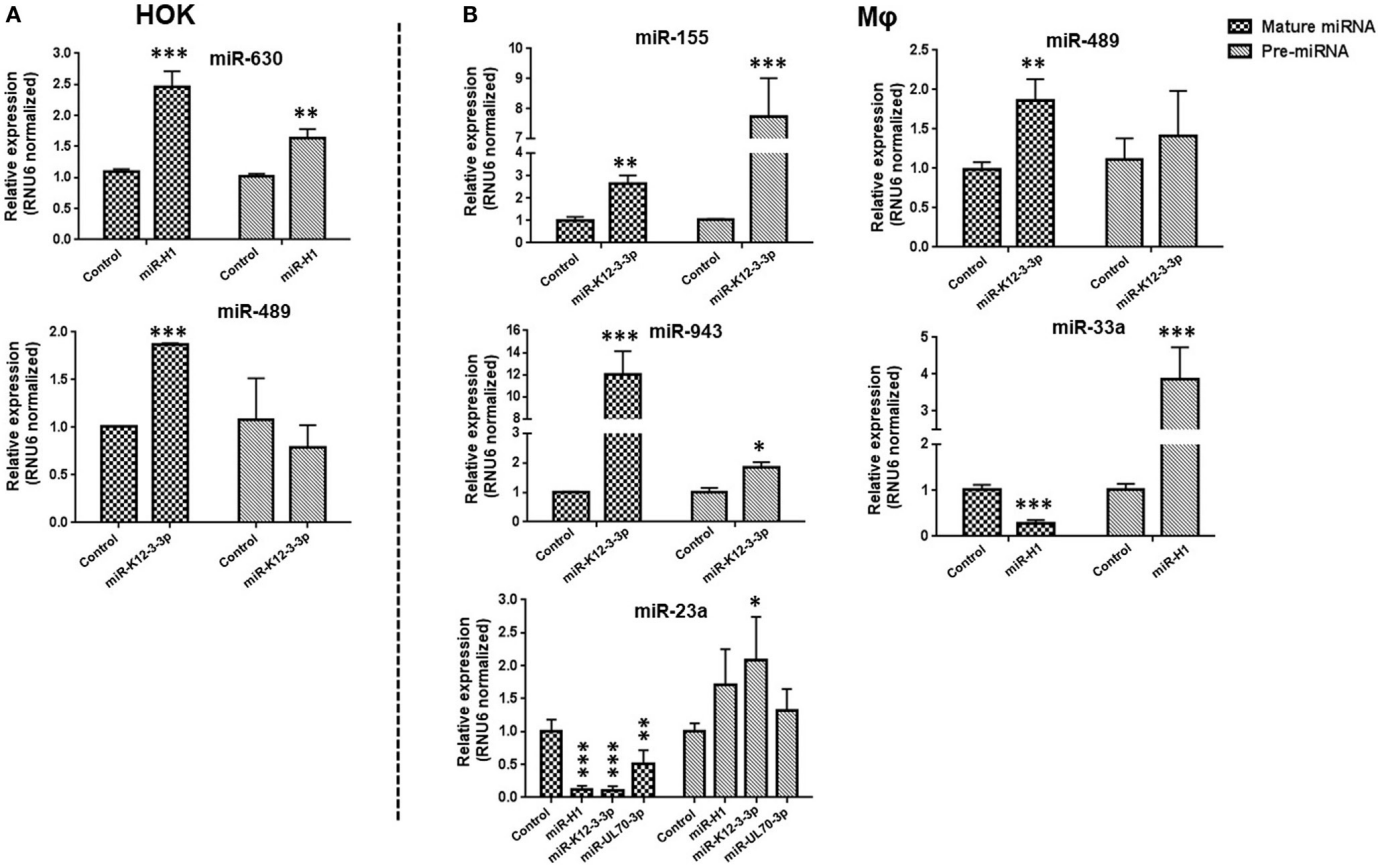
v-miR-mediated changes in cellular miRNAs occurs at transcription or post-transcription levels. Total RNA was isolated from human oral keratinocytes (HOK) and Mφ transfected with miR-H1, miR-K12-3-3p, miR-UL70-3p, or control mimic. Expression of precursor and mature miRNA of differentially expressed cellular miRNAs **(A)** miR-489, -630 in HOK and **(B)** miR-23a, -33a, -155, -489, and 943 in Mφ were determined by RT-qPCR. Histograms showing comparison of both pre- and mature miRNA profiles. Data are presented as mean ± SEM of four independent experiments. Student’s *t*-test was conducted to calculate *p*-values (*p < 0.05, **p < 0.01, ***p < 0.001).

From our miRNA microarray results, we observed that while expression of numerous miRNAs were altered by >1.5-fold change, others exhibit marked (>10-fold) alteration in expression levels. We next investigated whether these changes occur at the transcriptional and/or posttranscriptional level. To address this, expression of mature and pre-miRs of miR-489 and miR-630 (from HOK) and miR-23a, miR-33a, miR-155, miR-489, and miR-943 (from Mφ) was examined. In miR-H1-expressing HOK, we noticed that mature miR-630 expression corroborated with the corresponding pre-miR levels (Figure 4A; upper panel). In contrast, the expression of mature miR-489, but not pre-miR-489 levels were significantly induced in miR-K12-3-3p-transfected cells (Figure 4A; lower panel). For Mφ, we noted similar expression profiles of mature and precursor miRNA for miR-155, miR-943, and miR-489 (Figure 4B; upper and middle panel). The expression pattern of miR-23a and miR-33 exhibited an antagonistic relationship. However, pre-miR-23a induction is significant only in the presence of miR-K12-3-3p (Figure 4B; lower panel). Together, these results suggest that v-miR-mediated modulation of cellular miRNAs can occur at either the transcriptional or posttranscriptional level.

### v-miR Deregulated Cellular miRNAs Predominantly Target Cytoskeletal Organization, Endocytosis, and Signaling Pathways

Modulating functions of target cells is a strategy for viral tropism and persistence. Our results show that besides directly targeting transcripts, v-miRs can also modulate gene expression at the cellular miRNA level. To identify pathways impacted by v-miR-induced changes in host miRNAs, we employed *in silico* pathway analysis. Using the DIANA-miRpath pathway analysis algorithm, pathways targeted by cellular miRNAs and altered by v-miRs were identified. Many cellular pathways were identified for each cell type and viral miRNA examined. Table 1 provides a partial list of top 5–10 pathways identified in our pathway analysis.

**Table 1:** *In silico* pathway analysis of cellular miRNAs altered by v-miRs.

Viral miRNA (cell type)	Predicted pathways
HSV1 miR-H1 [human oral keratinocytes (HOK)]	Actin cytoskeleton Calcium signaling Focal adhesions MAPK pathway PI3K-Akt signaling pathway Wnt pathway

KSHV K12-3p (HOK)	Focal adhesions pathway Adherens pathway Actin cytoskeleton genes MAPK signaling pathway PI3K-Akt pathway

Human cytomegalovirus (HCMV) UL-70 (HOK)	MAPK gene Focal adhesions pathway Gap junction genes ERB pathway

HSV1 miR-H1 (Mφ)	Calcium signaling pathways Endocytosis pathway MAPK signaling pathway T cell receptor genes Wnt signaling pathway RNA transport genes PI3K-Akt genes TGF-β signaling pathway

KSHV K12-3p (Mφ)	Endocytosis Pathway MAPK Signaling Pathway PIK-Akt Pathway Protein Production in ER RNA Transport Genes Wnt Signaling Pathway

HCMV UL-70 (Mφ)	Apoptosis Chemokine Endocytosis pathway Erb pathway Osteoclast differentiation pathways PI3K-Akt pathway T-cell receptor genes

*Cellular miRNAs dysregulated by v-miRs in both HOK and Mφ were analyzed for the identification of cellular pathways. List of top 5–8 pathways are listed*.

For HOKs, the pathways affected by HSV1 miR-H1 related to the actin cytoskeleton, calcium signaling, focal adhesions, MAPK pathway, PI3K-Akt pathway, and Wnt signaling pathway. For KSHV K12-3p, the pathways related to focal adhesions, adherens, actin cytoskeleton, MAPK signaling, and PI3K-Akt pathway. For HCMV miR-UL-70, the pathways affected were associated with focal adhesions, gap junctions, MAPK signaling, and the Erb pathway. Together our pathway analysis suggests that the function of epithelial cell migration, adhesion, and transcription may be significantly affected.

For Mφ, the pathways affected by HSV1 miR-H1 related to calcium signaling, endocytosis, MAPK signaling, T-cell receptor, Wnt signaling, RNA transport, PI3K-Akt pathways, and TGF-β signaling pathways. For KSHV K12-3p, the pathways affected related to endocytosis, MAPK signaling, PIK-Akt signaling, protein production in ER, RNA Transport, and Wnt signaling. For HCMV UL-70, the pathways affected included apoptosis, chemokines, endocytosis, Erb signaling, osteoclast differentiation, PI3K-Akt, and T-cell receptor. Together, this analysis shows how the functionality of Mφ may be affected based on alterations to phagocytosis, endocytosis, and transcription. Of most interest was the possibility that HCMV UL-70 may alter or affect osteoclast differentiation.

### Viral miRs Directly Regulate Genes Involved in Endocytic and Intracellular Trafficking Pathways

**Figure 5:**
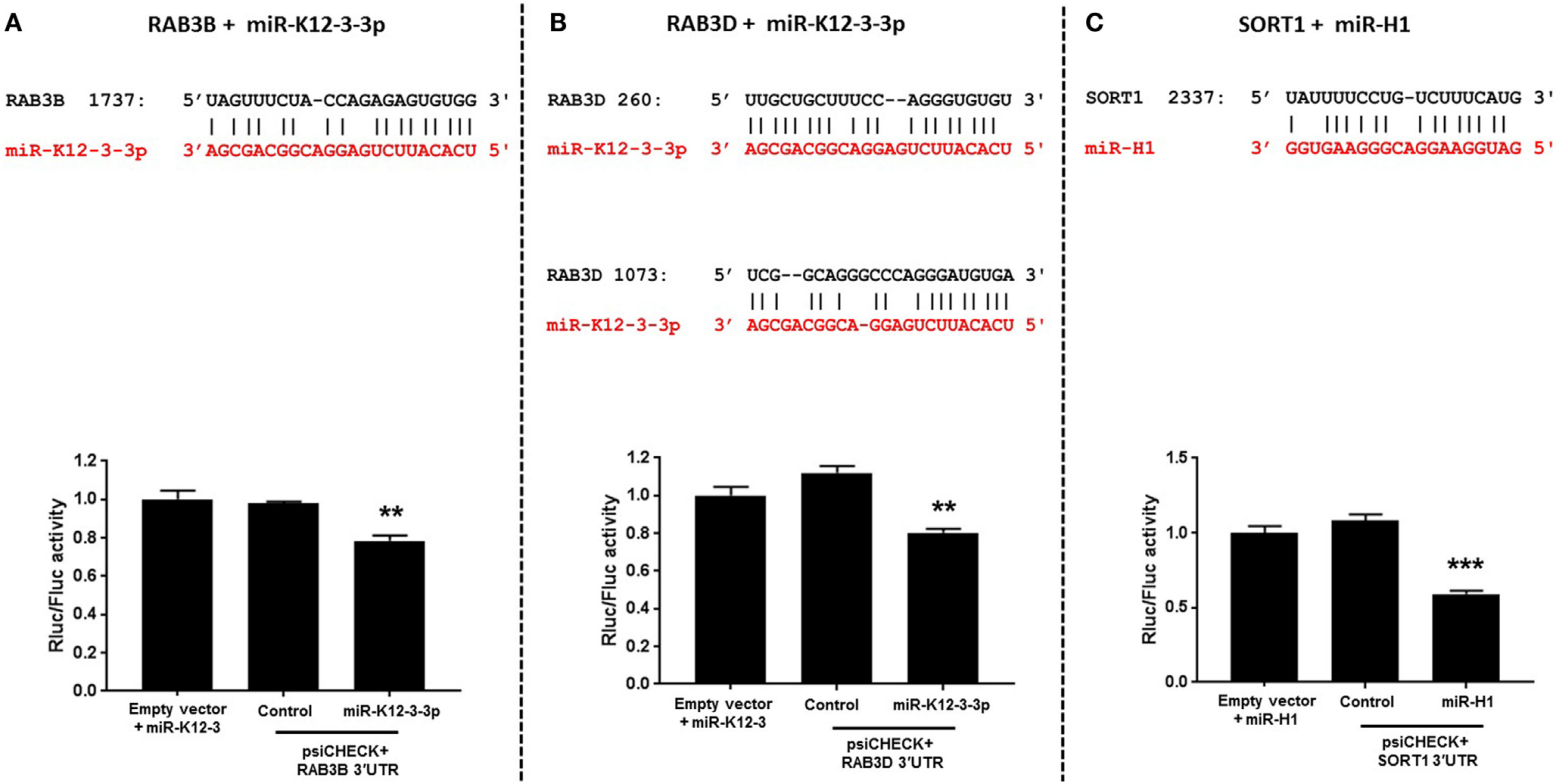
Viral miRNAs directly regulate genes involved in endocytosis. **(A–C)** Top panel shows the sequence alignment of the predicted miR-K12-3-3p and miR-H1 (in red) binding site on the target genes RAB3B, RAB3D, and SORT1 (in black). HEK293 cells were co-transfected with putative gene 3′UTR construct and with either miR-K12-3-3p, miR-H1, or control mimic. Renilla activity was normalized to firefly activity and the ratios subsequently normalized to empty vector transfected with viral miRNA mimic set as 1. **(A–C)** Histograms (bottom panel) shows impact of miR-H1 on normalized renilla activity compared to experimental controls. Error bars represent mean ± SEM from three biological replicates. *p*-Values were calculated using Student’s *t*-test (**p < 0.01; ***p < 0.0001).

Endocytosis and cell trafficking are critical cellular pathways targeted by viruses to successfully evade immune responses and persist within the host. We, therefore, examined the genes with putative v-miRNA binding sites using the Vir-Mir database (see text footnote 3). Three genes namely RAB3B, RAB3D (miR-K12-3-3p target), and sortilin 1 (miR-H1 target), identified in our *in silico* screening, were selected for further binding site validation. Figure 5 (upper panel) shows the sequence alignment of v-miR and their respective predicted targets. To confirm these interactions, we cloned the 3′UTR of these genes encompassing the predicted v-miR binding site into a dual luciferase expressing construct, psiCHECK2. As shown in Figures 5A,C, single binding sites for RAB3B and SORT1 were identified while two miR-K12-3-3p sites were identified in RAB3D (Figure 5B). These constructs were co-transfected with v-miRs or control mimic. After 36 h, cell lysates were prepared and analyzed for renilla and firefly luciferase activity. Our results show that compared to control mimic or negative control transfected cells, significantly reduced renilla activity (normalized to firefly) was observed for RAB3B, RAB3D (both miR-K12-3-3p) and SORT1 (miR-H1) constructs (Figures 5A–C; lower panel). This strongly suggests that v-miRs targets endocytosis pathway through direct downregulation of genes linked to the pathway.

### miR-H1 and miR-K12-3-3p but Not miR-UL70-3p Attenuates Phagocytosis by Mφ

**Figure 6:**
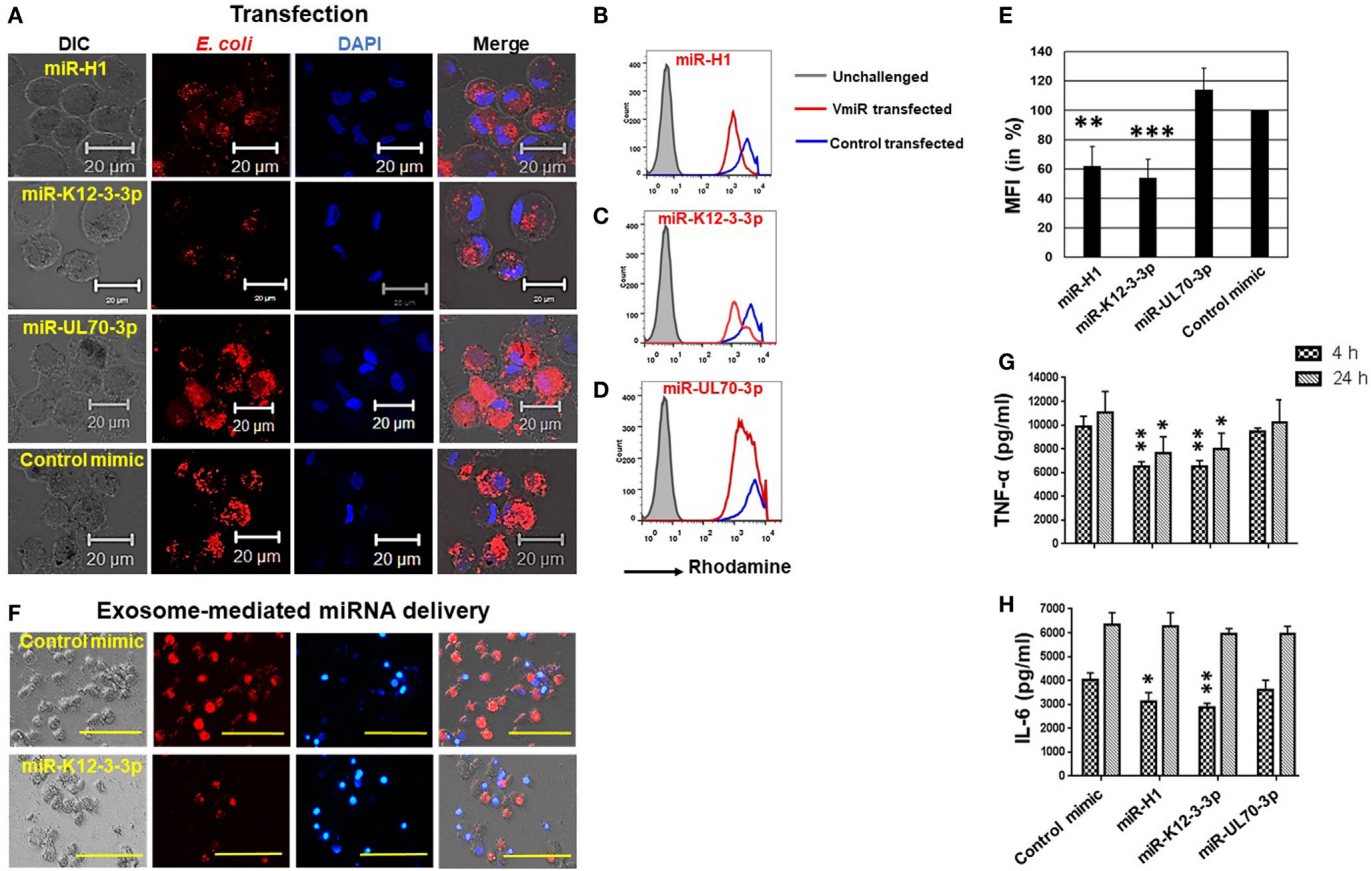
Viral miRNAs attenuate phagocytosis in macrophages. Macrophages were transfected with miR-H1, miR-K12-3-3p, miR-UL70-3p, or control mimics. After 36 h, phagocytosis assays were performed by incubating cells with rhodamine-labeled heat-killed *E. coli* bioparticles for 1 h. **(A)** Representative confocal images showing reduced internalization of *E. coli* in miR-H1 and miR-K12-3-3p transfected cells compared to control mimics. Scale bar, 20 µm. Quantitative analysis of the impact of phagocytosis was assessed by flow cytometry. Histograms showing comparison of rhodamine florescence in **(B)** miR-H1, **(C)** miR-K12-3-3p, and **(D)** miR-UL70-3p transfected Mφ with the control mimics. **(E)** Histograms showing percent mean florescence intensity changes in control or v-miR transfected Mφ. Data are presented as mean ± SEM from five donors. **(F)** Exosomes isolated from human oral keratinocytes were transfected with miR-K12-3-3p or control mimic using Exo-Fect as per manufacturer’s instruction and then incubated with U937 Mφ. After 24 h, phagocytosis assay was performed by incubating cells with *E. coli* bioparticles for 4 h. Representative florescence microscope images shows attenuation of *E. coli* uptake (red signals) in cells incubated with miR-K12-3-3p carrying exosomes compared to control. Scale bar, 100 µm. Supernatant levels of **(G)** TNF-α and **(H)** IL-6 were assayed after 4 and 24 h in supernatants of v-miR or control transfected primary Mφ by ELISA. Student’s *t*-test was conducted to calculate *p*-values (*p < 0.05, **p < 0.01, ***p < 0.001).

Human herpesvirus persists as a subclinical, recurrent infection for the lifetime of an individual. This requires successful evasion of various host immunosurveillance mechanisms. Although a role of HHV in augmenting various diseases has been proposed, there is little information on how they modulate immune responses. Phagocytosis is a process by which immune cells recognize, internalize, and degrade antigens. This is a key step in antigen presentation, another feature of the Mφ, a potent antigen presenting cell. To investigate the impact of v-miR on phagocytosis, we used heat killed and rhodamine labeled *E. coli* as the model pathogen. Upon uptake by Mφ, the rhodamine labeled *E. coli* will fluoresce within the acidic pH of the phagolysosome. Day 7 differentiated Mφ were transfected with miR-K12-3-3p, miR-H1, miR-UL-70-3p, or control mimics for 24 h. Cells were then challenged with *E. coli* and incubated for 2 h. Confocal imaging shows that miR-K12-3-3p and miR-H1 but not miR-UL-70-3p attenuated phagocytosis of *E. coli* by Mφ compared with control mimics (Figure 6A). Flow cytometry analysis confirms reduced florescence in miR-K12-3-3p and miR-H1 transfected cells compared to control as reflected by a left shift in mean florescence intensity (MFI; Figures 6B–D). To quantitate the attenuation in bacterial uptake, we analyzed percent change in MFI and observed ~50% and ~40% reduction in miR-K12-3-3p and miR-H1 transfected Mφ (Figure 6E) respectively, compared to controls. This suggests that v-miR-mediated changes in host miRNA have functional consequences.

To further validate that exosome-packaged v-miRs are functional, we collected exosomes from HOK and transfected them with miR-K12-3-3p or control mimic using Exo-Fect. These v-miR overexpressing exosomes were incubated with U937 Mφ. Phagocytosis assays were performed using rhodamine labeled *E. coli* as discussed above. Compared to control mimic, attenuated uptake of *E. coli* was observed in Mφ incubated with exosomes that delivered miR-K12-3-3p (Figure 6F). These results further confirm that v-miRs packaged in exosomes are functional and delivery of v-miRs through HOK-derived exosomes can impair cellular functions of Mφ.

### Aberrant Cytokine Profiling Exhibited by miR-H1 and miR-K12-3-3p Transfected Mφ Challenged with *E. coli*

The host innate immune response is highly dependent on the capability of Mφ to recognize and initiate inflammatory responses against antigens. Upon antigen recognition, Mφ profusely secrete a wide array of pro-inflammatory cytokines that is tightly coupled to phagocytosis. Since we observed a significant impact on phagocytosis of *E. coli* in miR-K12-3-3p and miR-H1 transfected cells, we examined the impact of v-miRs on secretion of the pro-inflammatory cytokines TNF-α and IL-6. Supernatants from miR-K12-3-3p, miR-H1, miR-UL70-3p, or control mimic transfected and *E. coli* challenged Mφ were collected after 4 and 24 h. ELISA results show that compared to control, supernatant levels of TNF-α and IL-6 were significantly lower in miR-K12-3-3p and miR-H1 but not UL-70-3p transfected cells (Figures 6G,H). These results corroborated with the reduced phagocytosis activity observed.

To gain a better understanding of cytokine secretion, we performed multiplex analysis of 23 different cytokines (including TNF-α and IL-6). Our results show that compared to control mimics, v-miR transfected Mφ challenged with *E. coli* exhibit remarkably different cytokine/chemokine profiles. In general, we noticed downregulation of various pro- and anti-inflammatory cytokines. As expected, the levels of different cytokines vary with time. Secreted IL-1β, IL-6, IL-8, MCP-1, MCP-3, MIP-1α, MIP-1β, RANTES, TGF-β, and TNF-α levels were detected at both early and late time points while, granulocyte/macrophage colony stimulating factor (GM-CSF), IFN-γ, IL-10, IL-12 p40, IL-12 p70, TGF-α, and TNF-β levels were predominantly detected at the later time point. On the other hand, IL-2, IL-4, IL-7, IL-9, IL17A, and sCD40L were not detected at any of the time points examined.

**Figure 7:**
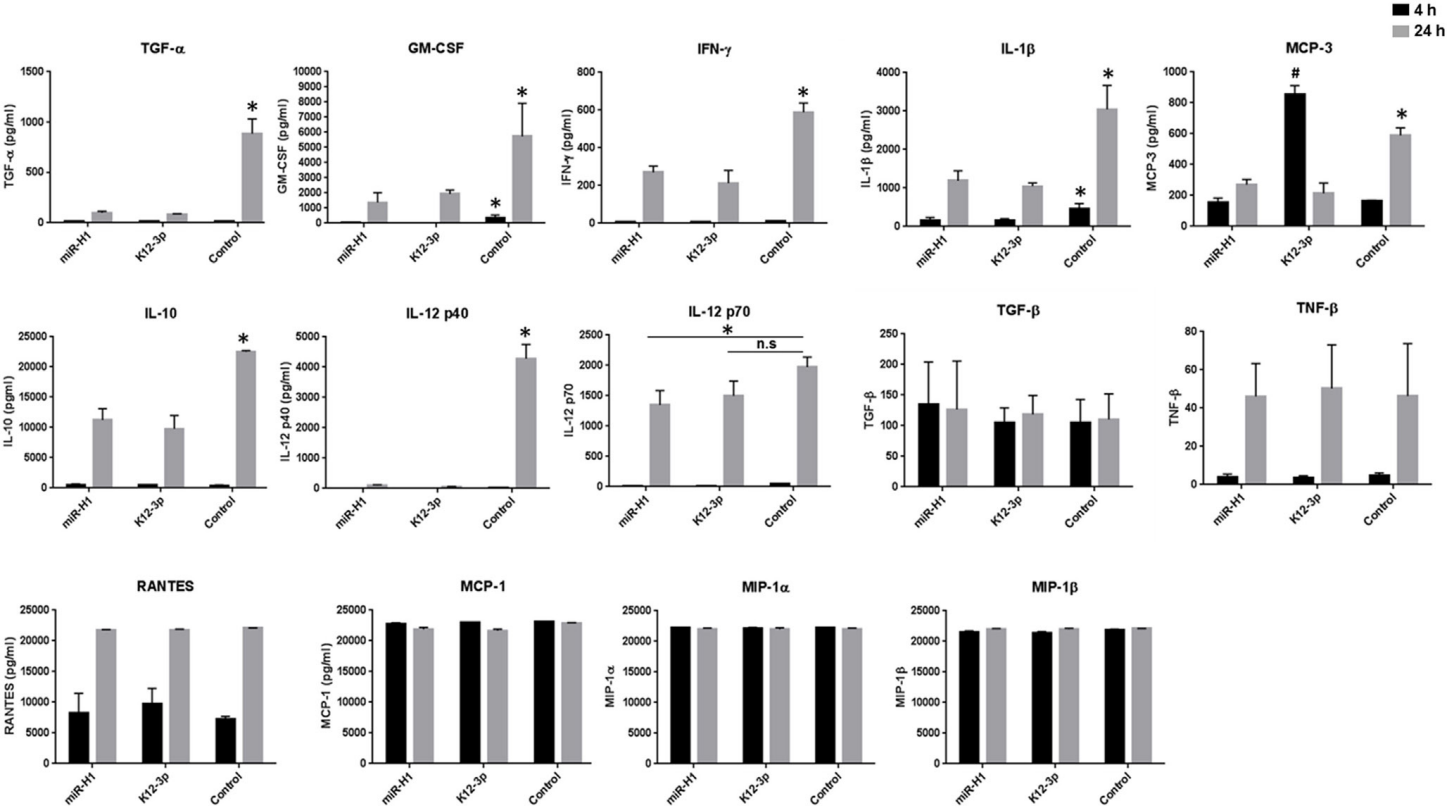
v-miRs transfected Mφ exhibit aberrant cytokine secretion profiles. Day 7 differentiated Mφ were transfected with miR-H1, miR-K12-3-3p, or control mimics. After 24 h, cells were challenged with *E. coli* for 4 and 24 h and supernatants were collected. Secreted levels of 22 different cytokine/chemokine were analyzed by multiplex bead array. Data are presented as mean ± SEM from four independent donors. Student’s *t*-test was conducted to calculate *p*-values (*p < 0.05, **p < 0.01, ***p < 0.001).

Among the cytokines detected, we noticed significant downregulation of GM-CSF and IL-1β at 4 h and 24 h post-challenge (Figure 7). Interestingly, a much more pronounced effect was observed at the later time point (24 h) for GM-CSF, IFN-γ, IL-1β, IL-10, IL-12 p40, IL-12 p70, MCP-3, and TGF-α levels. Due to high sensitivity of the multiplex assay, levels of MIP-1α, MIP-1β, MCP-1 (Figure 7), IL-6, IL-8, TNF-α (data not shown) reached the limit of saturation at both twofold and sixfold dilution. We also noticed differential cytokine responses in miR-H1 and miR-K12-3-3p transfected Mφ. For instance, significantly increased MCP-3 levels were detected at the early time point in miR-K12-3-3p-transfected cells while miR-H1 transfection had no impact compared to control. Increased, albeit not significant, RANTES levels were observed at the early time point in miR-K12-3-3p transfected cells. Levels of TNF-β and TGF-α did not show any significant differences at both the time points (Figure 7). Table 2 summarizes the modulation of various cytokines. Overall, these results clearly support an immunomodulatory impact of v-miRs on innate immune response by Mφ that can likely perturb downstream immune cell activation and priming.

**Table 2:** Summary list showing the changes in the secreted cytokine/chemokine levels in supernatants of Mφ challenged with *E. coli* for 4 and 24 h.

Altered secretion Up (↑)/down (↓)/no change (NC)		
**Cytokines/chemokines**	**4-h stimulation**	**24-h stimulation**
Granulocyte/macrophage colony stimulating factor	↓	↓
IL-1β	↓	↓
IL-6	↓	↓
IL-8	↓	↓
IL-10	NC	↓
IL-12 p40	NC	↓
IL-12 p70	↓	↓
IFN-γ	NC	↓
MCP-3	↑ (miR-K12-3-3p only)	↓
TGF-α	NC	↓
TNF-α	↓	↓

## Discussion

Despite the fact that the presence of herpesviruses in the oral cavity has been known for some time, essentially nothing is known regarding the role of viral factors in the pathogenesis of oral inflammatory diseases. In separate cohorts of subjects with pulpitis (24) and periodontitis (Naqvi et al., submitted), our group observed higher levels of herpesvirus miRNAs. Increased prevalence of HSV1, HCMV and KSHV (encoding miRNAs evaluated in this study) has been reported in various oral diseases (1–5). In the present study, we demonstrate that viral miRNAs can impact the host miRnome in two key oral mucosal cells, *viz*., gingival keratinocytes and macrophages, and hence modulate host innate immune responses. Our results suggest that HHV encoded non-coding RNAs (v-miRs) produced during oral infection can profoundly impact host defense and likely exacerbate pathogenesis of oral inflammatory diseases primarily considered of bacterial origin.

Epithelial cells and myeloid cells (monocytes/Mφ/DCs) are two key targets for most herpesviruses. In order to maintain lifelong infection, herpesvirus must modulate specific host cell functions. They often achieve this by reprogramming host cells, making the cellular environment more permissible to viral replication and survival (35, 36). This is primarily achieved by modulating diverse genes and cellular pathways. Therefore, identifying individual viral components associated with modulation of host pathways will be vitally important in development of targeted antiviral strategies. In this study, we examined whether v-miRs alter host cellular miRNAs profiles and evaluated their impact on key functions of each cell type. We selected three v-miRs representing each member of herpesvirus subfamily, *viz*., miR-H1, miR-K12-3-3p, and miR-UL-70-3p to examine the impact on the expression of cellular miRNAs profiles. Our results highlight altered expression of numerous cellular miRNAs in both HOK and Mφ transfected with miR-H1 and miR-K12-3-3p. Conversely, we noted modulation of very few cellular miRNAs in miR-UL70-3p transfected cells. Alteration in the expression of cellular miRNAs is a potentially more robust strategy compared to the modulation of protein-coding transcripts because miRNAs can regulate hundreds of genes simultaneously thereby controlling multiple cellular functions. Unlike previous studies which were focused on the identification of viral and cellular mRNAs targets of v-miRs, we examined and confirmed that viral miRNAs can also modulate numerous host miRNAs. Thus, v-miRs can regulate viral mRNAs as well as host mRNA and miRNAs. Given their significant capacity to regulate hundreds of hosts’ mRNA and miRNAs, viral miRNA can significantly influence the host transcriptome and hence, cellular function such as immunity as we demonstrate in this study.

Changes in cellular miRNAs expression have been demonstrated in virus-infected cells. The role of v-miRs, however, was not explored. Induced expression of miR-155 and miR-21 has been associated with EBV and KSHV infections (37–39). We observed increased expression of miR-155-5p and miR-21-3p in Mφ by miR-K12-3-3p and miR-H1. These findings suggest that v-miR transfection somehow mimics viral infection regarding host miRNA profiles. Additionally, we show that miR-K12-3-3p-mediated overexpression of mature miR-155-5p occur due to accumulation of pre-miR-155 suggesting that v-miRs can impact transcription of miRNAs. Similar correlation in the mature and precursor miRNA was also observed for miR-630, miR-943, miR-489 (Mφ). However, pre- and mature miRNAs levels of miR-23a and miR-33a did not corroborate. Thus, v-miRs-mediated changes can occur either at transcriptional or posttranscriptional levels. Further studies are required to dissect the underlying mechanisms of v-miR impact on the miRNA biogenesis pathway.

Interestingly, most of the cellular miRNAs exhibit v-miR-specific expression suggesting that v-miRs can regulate a unique repertoire of host miRNAs. For example, HOK transfected with miR-H1 and miR-K12-3 share only one host miRNA. This clearly indicates that v-miR target unique subsets of host miRNAs that likely deregulate associated but specific cellular pathways/functions. This is supported by the fact that unlike metazoan miRNAs, v-miRs sequences are poorly conserved even in closely related families indicating their functional divergence (40). Importantly, Dölken et al. (41) showed that v-miRs regulate genes with specific functions. For instance, KSHV encoded miRNAs repressed host targets involved in gene expression and regulation but such functional bias by EBV was not observed. This indicates that certain v-miRs have evolved to modulate specific host pathways. Our results showing changes in cellular miRNAs profiles by miR-H1 and miR-K12-3 reveal the novel role of v-miRs in modulating host cell functions and suggest their role in infection, establishment, and pathogenesis. Global pathway analysis of altered cellular miRNAs identifies several pathways including cell signaling, cell proliferation, endocytosis, viral infection, etc., indicating profound impact of v-miRs on cellular pathways through modulation of cellular miRNAs. This provides strong evidence that v-miR-mediated modulation of host miRNAs can subsequently and specifically impact the immune response. As such, alterations in cellular miRNA profiles may facilitate immune evasion.

The oral cavity is strategically positioned with Mφ capable of initiating innate immune responses and shaping adaptive immunity. Pathway analysis of v-miR altered cellular miRNAs identified several pathways that are closely associated with the uptake of particles including actin cytoskeleton, focal adhesions, adherens pathway, endocytosis pathway, PI3K-Akt signaling, etc. These pathways are pivotal for host cell functions and antiviral immune responses (42, 43). We further show that miR-K12-3-3p and miR-H1 directly bind genes involved in endocytosis and cellular trafficking and regulate their expression using a reporter assay. While miR-K12-3-3p targets RAB3A and RAB3B, miR-H1 regulates SORT1. Ras-associated binding (Rab) proteins are monomeric G-proteins that belong to the Ras superfamily and participate in vesicle formation and motility by membrane and protein trafficking (44). Among the four different isoforms of Rab, Rab3B, and Rab3D positively regulate exocytosis while Rab3A and Rab3C negatively regulate the process (45). Interestingly, besides these validated proteins, *in silico* analysis also identified various other genes linked to endocytic pathways as putative targets of these v-miRs. The significance of the endosomal pathway in herpesvirus entry, persistence, and immune evasion has been reported by various studies (43, 46, 47). In DC, siRNA based screening of 57 Rab GTPases identified the involvement of Rab3b/c-coated vesicles in cross antigen presentation (48). Rab3b/c co-localize with MHC I positive membranes in the perinuclear region. This is a key antiviral response where bystander DCs could cross-present exogenous viral Ag derived from infected and dying cells to secure priming of virus-specific CD8+ T cells (49, 50). Survival of HHV requires effective evasion of cytolytic CD8+ T cells which could be achieved by suppression of MHC I presentation or cross-presentation. Our recent transcriptome profiling show down-modulation of multiple RAB transcripts by miR-K12-3-3p (GEO Accession number GSE107005; Naqvi et al., submitted) and two of those RAB3B and RAB3D are now confirmed as novel targets in this study. The significance of blocking Ag presentation by MHC is highly evident in HHV with numerous examples of proteins and v-miRs targeting various components of this pathway (51–53). Sortilin 1 (or neurotensin receptor 3), a novel miR-H1 target identified in this study, is a Vps10p domain containing, multifunctional transmembrane proteins that triggers internalization of various ligands by endocytosis and sorts ligands between several intracellular compartments (54, 55). Sort1 binds to granulin and facilitates presentation of CpG DNA (including herpesviral genome) to endosomal TLR9, and hence triggers pro-inflammatory immune response upon viral invasion (56). Downregulation of Sort1 by miR-H1 thus allows cell infection and evasion of host immune responses. Interestingly, the expression of Sort1 is induced in bacterial infection suggesting its role in activation of innate immunity against diverse pathogens (57). The targeting of novel components of endocytic pathway by two different herpesvirus miRs signifies the importance of this pathway in herpesvirus survival and immune evasion.

Our results show that Mφ transfected with miR-H1 or miR-K12-3-3p exhibit attenuated phagocytosis of the Gram-negative bacteria *E. coli*. Because phagocytosis is a complex process that apart from receptors, predominantly involves a similar set of proteins to mediate the process, it is plausible that phagocytosis of Gram-positive bacteria may also be adversely impacted by v-miRs. This is supported by our global pathway analysis of cellular miRNAs altered by v-miRs. This suggests that several pathways associated with phagocytosis may be modulated either directly by v-miRs or indirectly by v-miR-mediated changes in cellular miRNAs. In patients with inflamed pulps or periodontitis, we noticed induced levels of several herpesvirus encoded v-miRs indicating their role in inflammation and disease pathogenesis [(24); Naqvi et al., submitted].

The oral cavity is an ecological niche of diverse microbial species. Several bacterial and viral species are known to coexist in the oral niche. Both Gram-positive and Gram-negative bacteria are recognized as causal agents for various oral diseases while herpesviruses are the predominant family of viruses detected in such infections. There are multiple reports providing evidence of a close association of herpesvirus with the development and severity in apical periodontitis, periapical lesions, pulpitis, etc. For instance, Ferreira et al. (3) reported that 48% of apical abscesses samples had KSHV infections, whereas Chen et al. (58) detected HCMV in 29% of the patients with similar disease. Thus, higher expression of v-miR observed in patients with oral inflammation may facilitate survival of oral bacteria.

Recognition of pathogen associated molecular patterns by cells initiate multiple signaling cascades that leads to activation of innate responses. Secretion of cytokines/chemokines not only allows cells to immediately respond to incumbent threats but also trigger activation and chemotaxis of other cells of the immune system. Our multiplex analysis of 23 different cytokines/chemokines revealed significant changes in secreted levels in miR-H1 or miR-K12-3-3p transfected Mφ. These changes were time responsive, as we noticed 6 and 11 analytes that were impacted at early (4 h) and late (24 h) time points, respectively, including both pro- and anti-inflammatory cytokines. v-miR mediated reduction in key cytokine levels may have a profound impact on the intercellular communication during the inflammatory response. Indeed, reduced but not abolishing immune responses is a feature of herpesviruses which allows them to persist as lifelong infection in a host with functional immunity. For instance, EBV mediated induction of cellular miR-155 leads to suppression of pro-inflammatory NFκB signaling (38). This facilitates in maintaining latency cycle of herpesvirus and hence long-term persistence. In our miRnome profiling, we also noticed higher expression of mature miR-155 (and pre-miR-155) in miR-K12-3-3p-transfected Mφ. Reduced cytokine/chemokine secretion upon bacterial challenge can be attributed to induced miR-155 levels by miR-K12-3-3p. Evidently, immune subversion is closely associated with clinical manifestation of herpesvirus infection as symptomatic infection is exhibited in immunocompromised individuals (1, 2, 51, 59).

Inherent functional plasticity of Mφ allows them to elicit adept immune responses. Depending on the activation status, Mφ phenotype can be broadly classified as classically (M1) or alternatively (M2) activated (60, 61). M1 Mφ are primarily involved in Th1 responses, lymphokine production, and degradation of intracellular pathogens, whereas M2 Mφ trigger Th2 responses, immunotolerance, and tissue remodeling (61). Multiple lines of evidence highlight the association of HHV in manifesting oral inflammatory diseases (1–5, 58). HHV have evolved elegant mechanisms to subvert host immunity and the role of v-miRs in this context is emerging (62–65). In our multiplex cytokine/chemokine analysis, we observed significant downregulation of multiple cytokines including TNF-α, IFN-γ, IL-6. These cytokines are associated with Th1 polarization and v-miR mediated reduction of these cytokines can modulate Th-skewing which can impact viral detection and activation of efficient adaptive immune responses by T cells. Indeed, these cytokines are evidently categorized as antiviral because of their capacity to mediate Th1 polarization. Another key cytokine that was significantly reduced in the multiplex analysis is GM-CSF, a pro-inflammatory cytokine produced at high levels in response to LPS or TNF-α/IL-1 (66, 67). GM-CSF increases mobility of monocytes, primes them for inflammatory response and induces ROS generation. Mφ exposed to GM-CSF are polarized to inflammatory status (M1) by restricting M-CSF signaling. Reduced GM-CSF production by miR-H1 and miR-K12-3-3p transfected cells can, therefore, maintain a low inflammatory status thereby maintaining a microenvironment that will favor immune evasion by the virus, a key feature of long-term latency requirement herpesviruses. These results suggest that v-miRs can evade hosts’ antiviral responses, in part, by altering the cytokine profiles. Taken together, these findings support the possibility that induced levels of v-miRs during microbial challenge attenuates phagocytosis as well as cytokine/chemokine secretion by Mφ. In this regard, v-miRs can be key factor contributing to increased bacterial growth, and subsequent pathogenesis in oral tissues.

In conclusion, this study identifies novel molecular and functional mechanisms through which herpesvirus derived v-miRs can modulate host cell (HOK and Mφ) function. We show that overexpression of v-miRs alter expression of cellular miRNAs. These cellular miRNAs were predicted to regulate numerous cellular pathways related but not limited to cell movement, signaling pathways, endocytosis, cell survival, and proliferation, etc. v-miRs can modulate phagocytosis and innate immune response by Mφ and can thus act a critical player in shaping oral microbiome in oral disease. Taken together, these findings highlight a novel regulatory mechanism through which v-miRs can modulate host cell immune responses and thus allow viruses to maintain lifelong infection. Our results provide insights into the role of v-miRs in the pathogenesis of oral inflammatory disease that can be potentially employed to devise targeted strategies aimed at the prevention and treatment of herpes viral infections.

## Author Contributions

AN, JS, and AS performed the experiments; AN, DS, and SN analyzed the data. AN and SN conceived the study and wrote the manuscript.

## Conflict of Interest Statement

The authors declare that the research was conducted in the absence of any commercial or financial relationships that could be construed as a potential conflict of interest.

## Footnotes

^1^http://www.mirbase.org/.

^2^http://diana.imis.athena-innovation.gr/DianaTools/index.php?r=mirpath/index.

^3^http://alk.ibms.sinica.edu.tw

## Supplementary Material

Figure S1 Genomic DNA was isolated from the primary human oral keratinocytes and Mφ before being utilized for subsequent experiments. PCR was performed using virus genome specific primers. Agarose gel electrophoresis showing human cytomegalovirus (HCMV) and HSV1 negative cultures. Isolation, PCR, and positive controls show expected amplicons.

Figure S2 Impact of v-miR transfection on cell viability. human oral keratinocytes and Mφ were transfected with miR-H1, miR-UL70-3p, and miR-K12-3-3p and cell viability was assessed by MTS assay 36 h posttransfection. Data are presented as mean ± SEM from four independent donors.

Figure S3 Cell type specific changes in the cellular miRNAs were assessed for **(A)** miR-H1, **(B)** miR-K12-3-3p, and **(C)** miR-UL70-3p transfected human oral keratinocytes and Mφ. Venn diagram showing the distribution of unique and overlapping altered cellular miRNAs.
