# Supplementary figures and images for: Viral miRNAs Alter Host Cell miRNA Profiles and Modulate Innate Immune Responses
Source: Front Immunol. 2018 Mar 6;9:433. doi: 10.3389/fimmu.2018.00433 (PMC5845630; doi:10.3389/fimmu.2018.00433)

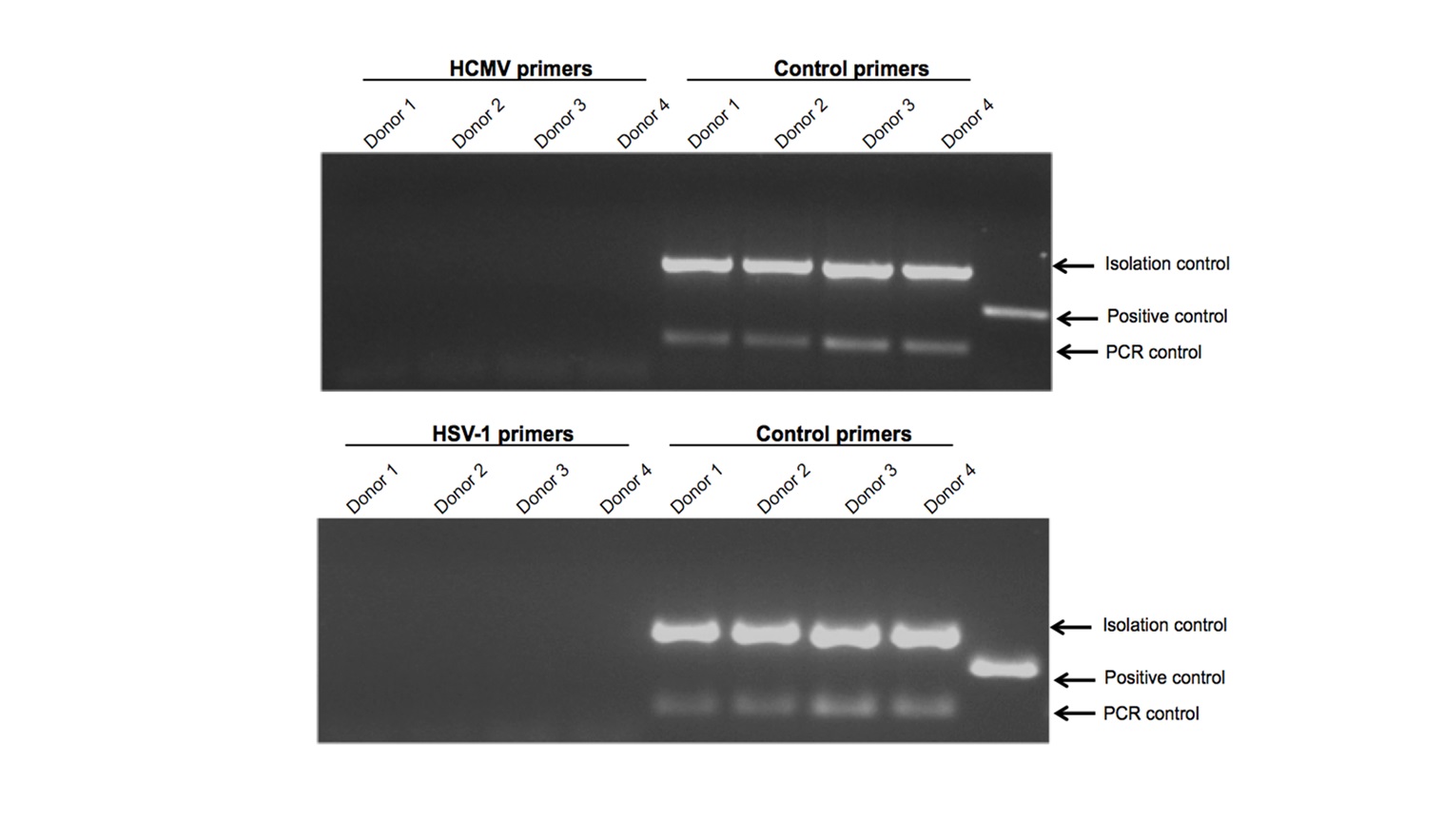

Supplement: Figure S1 — Genomic DNA was isolated from the primary human oral keratinocytes and Mφ before being utilized for subsequent experiments. PCR was performed using virus genome specific primers. Agarose gel electrophoresis showing human cytomegalovirus (HCMV) and HSV1 negative cultures. Isolation, PCR, and positive controls show expected amplicons. [file image_1.jpeg]

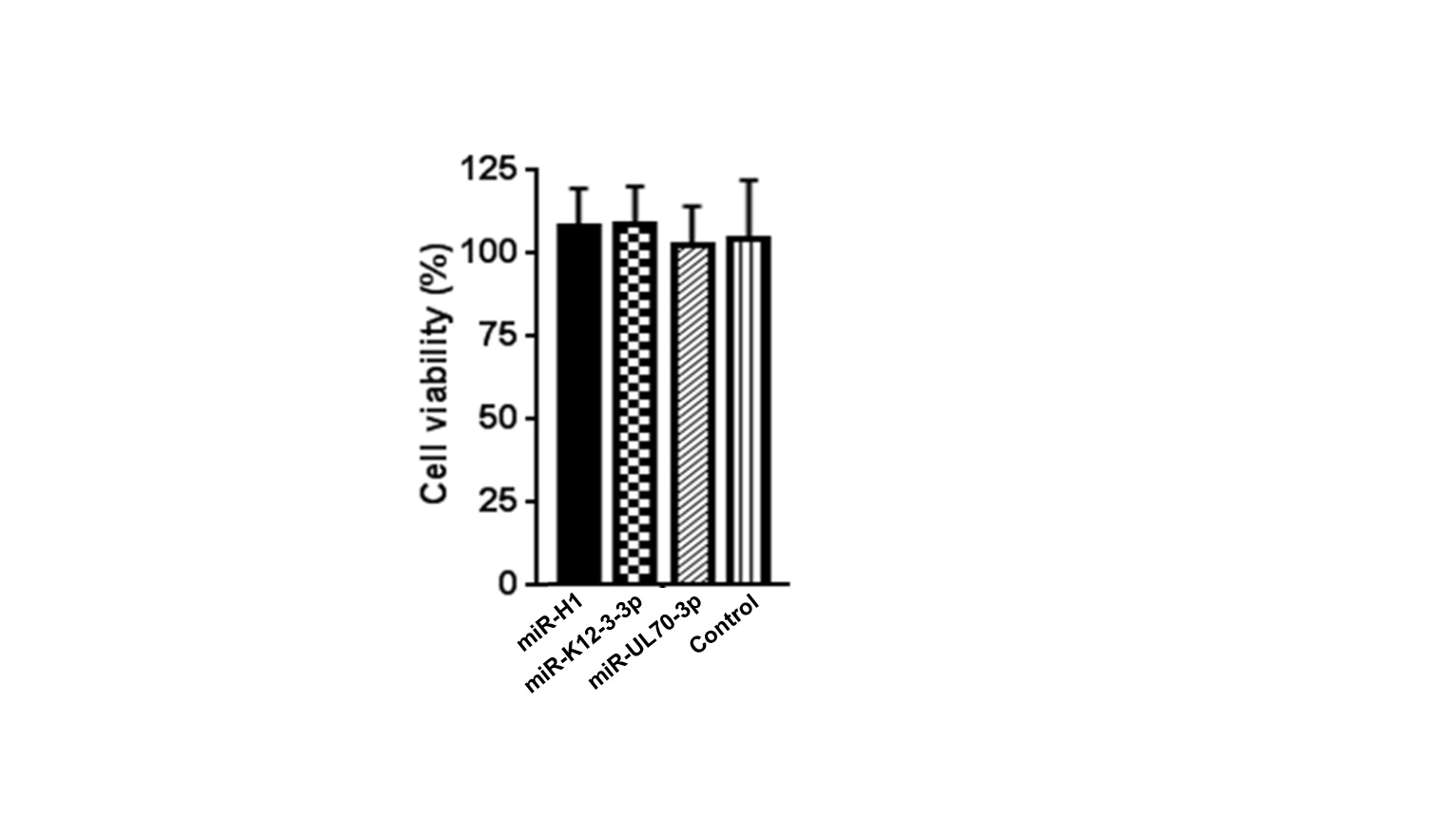

Supplement: Figure S2 — Impact of v-miR transfection on cell viability. human oral keratinocytes and Mφ were transfected with miR-H1, miR-UL70-3p, and miR-K12-3-3p and cell viability was assessed by MTS assay 36 h posttransfection. Data are presented as mean ± SEM from four independent donors. [file image_2.jpeg]

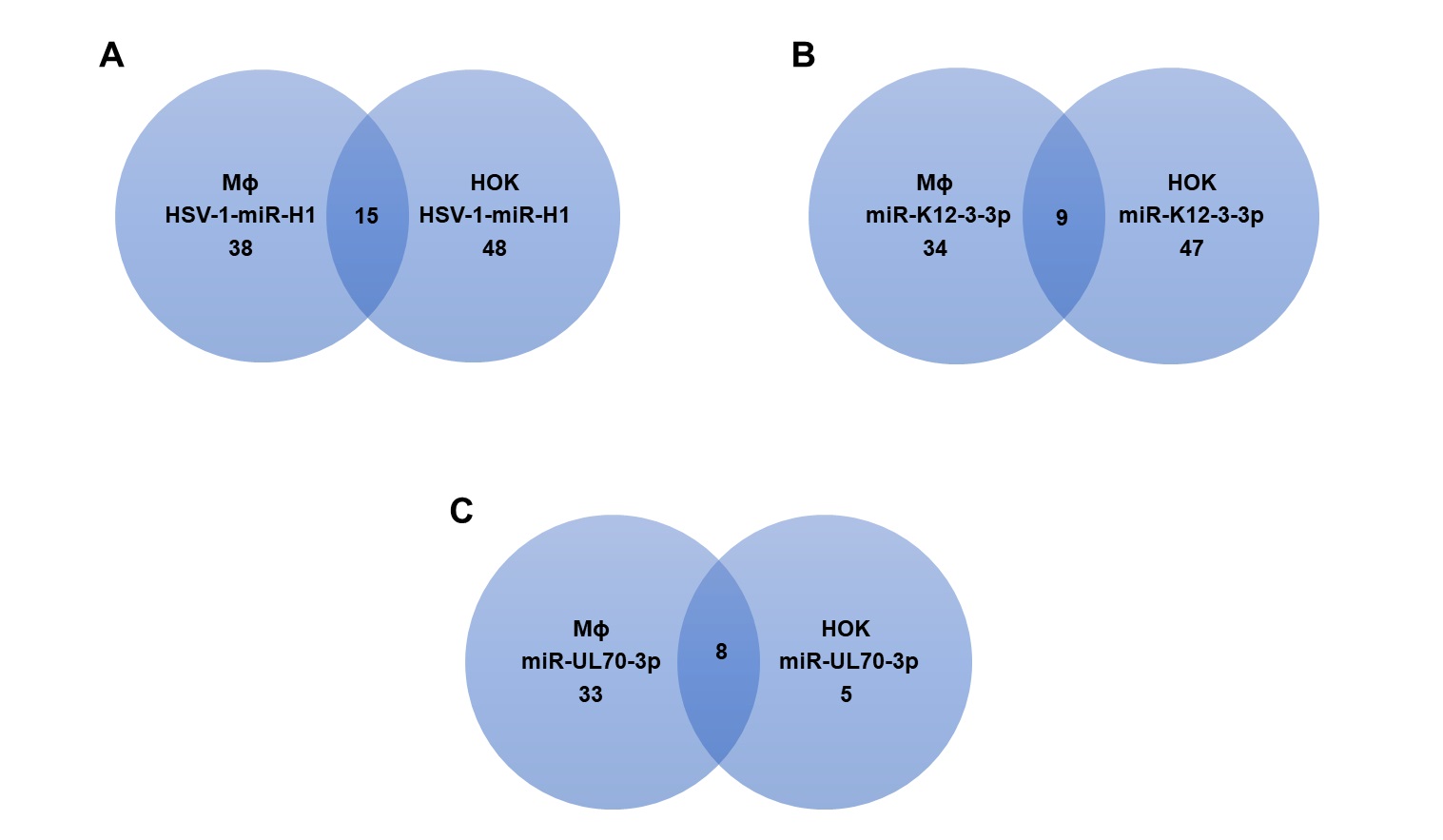

Supplement: Figure S3 — Cell type specific changes in the cellular miRNAs were assessed for (A) miR-H1, (B) miR-K12-3-3p, and (C) miR-UL70-3p transfected human oral keratinocytes and Mφ. Venn diagram showing the distribution of unique and overlapping altered cellular miRNAs. [file image_3.jpeg]
