# Supplementary material for: Viral miRNAs Alter Host Cell miRNA Profiles and Modulate Innate Immune Responses
Source: Front Immunol. 2018 Mar 6;9:433. doi: 10.3389/fimmu.2018.00433 (PMC5845630; doi:10.3389/fimmu.2018.00433)
Supplement: Supplementary file 4 [file table_1.doc]

| **Annotation** | **Fold change** |
| --- | --- |
| hsa-miR-204-3p° | 4.7564 |
| hsa-miR-4426° | 18.6008 |
| hsa-miR-4436b-3p° | 8.7163 |
| hsa-miR-4657° | 10.5757 |
| hsa-miR-4795-5p° | 9.2380 |
| hsa-miR-4450° | 14.1163 |
| hsa-miR-483-5p° | 1.6509 |
| hsa-miR-4669° | 3.5591 |
| hsa-miR-4764-5p° | 1.8408 |
| hsa-miR-630° | 1.8080 |
| hsa-miR-7-5p° | 1.6907 |
| hsa-miR-4497° | 1.5515 |
| hsa-miR-5006-3p° | 11.4094 |
| hsa-miR-21-3p° | 1.5333 |
| hsa-miR-33a-5p° | 0.6767 |
| hsa-miR-513a-3p | 4.2979 |
| hsa-miR-4484 | 1.3773 |
| hsa-miR-423-5p | 1.3391 |
| hsa-miR-1249 | 0.8076 |
| hsa-miR-3202 | 1.2767 |
| hsa-miR-425-5p | 0.8174 |
| hsa-miR-197-3p | 0.8424 |
| hsa-miR-3178 | 1.4821 |
| hsa-miR-4288 | 0.6323 |
| hsa-miR-4530 | 1.3383 |
| hsa-miR-26a-2-3p | 1.2181 |
| hsa-miR-423-3p | 0.8193 |
| hsa-miR-23a-3p | 0.6676 |
| hsa-miR-371b-5p | 1.6388 |
| hsa-miR-664a-3p | 0.8527 |
| hsa-miR-1246 | 1.5263 |
| hsa-miR-1913 | 0.8198 |
| hsa-miR-4456 | 0.7395 |
| hsa-miR-4800-3p | 1.4045 |
| hsa-miR-877-5p | 0.8526 |
| hsa-miR-1290 | 1.4303 |
| hsa-miR-4787-5p | 1.5729 |
| hsa-miR-3940-5p | 1.4954 |
| hsa-miR-4644 | 1.2295 |
| hsa-miR-665 | 1.3535 |
| hsa-miR-1469 | 1.1524 |
| hsa-miR-3158-5p | 1.4276 |
| hsa-miR-338-3p | 0.8478 |
| hsa-miR-4521 | 0.6436 |
| hsa-miR-4531 | 1.2719 |
| hsa-miR-4646-3p | 0.8645 |
| hsa-miR-4659b-3p | 0.8865 |
| hsa-miR-4708-3p | 1.6294 |
| hsa-miR-5196-3p | 0.8521 |
| hsa-miR-718 | 0.7895 |
| hsa-miR-874 | 1.2699 |
| hsa-miR-151a-5p | 0.7818 |
| hsa-miR-371b-3p | 0.8837 |

**Table S1.** List of differentially expressed cellular miRNAs in miR-H1 transfected HOK. Fold change values compared with control mimic are provided. MiRNAs with ‘°’ denotes FDR (Benjamini Hochberg Test) adjusted significance of p<0.05.

| **Annotation** | **Fold change** |
| --- | --- |
| hsa-miR-489° | 253.6377 |
| hsa-miR-4659b-3p° | 23.1735 |
| hsa-miR-1184° | 60.6539 |
| hsa-miR-15a-3p° | 4.0309 |
| hsa-miR-943° | 18.4791 |
| hsa-miR-1321° | 4.4890 |
| hsa-miR-4451° | 2.4445 |
| hsa-miR-3165° | 3.0411 |
| hsa-miR-21-3p° | 1.6425 |
| hsa-miR-365b-5p° | 1.4143 |
| hsa-miR-382-3p | 1.3381 |
| hsa-miR-9-5p | 1.4071 |
| hsa-miR-200b-5p | 1.2591 |
| hsa-miR-4288 | 0.7883 |
| hsa-miR-4423-5p | 0.8663 |
| hsa-miR-29c-3p | 1.1885 |
| hsa-let-7f-1-3p | 1.1436 |
| hsa-miR-214-3p | 1.1830 |
| hsa-miR-29b-3p | 1.2192 |
| hsa-miR-423-3p | 0.8535 |
| hsa-miR-4297 | 0.8653 |
| hsa-miR-4478 | 0.8254 |
| hsa-miR-4725-5p | 0.1675 |
| hsa-miR-5004-5p | 1.2661 |
| hsa-miR-600 | 1.1665 |
| hsa-miR-1285-3p | 1.1462 |
| hsa-miR-3620-3p | 1.1694 |
| hsa-miR-518e-5p/hsa-miR-519a-5p/hsa-miR-519b-5p/hsa-miR-519c-5p/hsa-miR-522-5p/hsa-miR-523-5p | 1.2053 |
| hsa-let-7d-5p | 1.1372 |
| hsa-miR-1915-3p | 1.1379 |
| hsa-miR-29a-3p | 1.1831 |
| hsa-miR-26b-3p | 1.1224 |
| hsa-miR-5100 | 1.1465 |
| hsa-miR-4695-3p | 1.1473 |
| hsa-miR-138-2-3p | 1.1535 |
| hsa-miR-19b-3p | 1.1270 |
| hsa-miR-204-3p | 0.7394 |
| hsa-miR-1260a | 1.1529 |
| hsa-miR-1304-5p | 0.8871 |
| hsa-miR-205-3p | 1.2212 |
| hsa-miR-210 | 0.8761 |
| hsa-miR-302a-3p | 0.7776 |
| hsa-miR-31-3p | 1.1313 |
| hsa-miR-431-5p | 0.8802 |
| hsa-miR-4511 | 0.6162 |
| hsa-miR-4797-5p | 1.1499 |
| hsa-miR-5006-3p | 1.1679 |
| hsa-miR-552 | 0.6978 |
| hsa-miR-1264 | 1.1474 |
| hsa-miR-3182 | 1.1221 |
| hsa-miR-3676-5p | 1.1246 |
| hsa-miR-4292 | 1.1345 |
| hsa-miR-29a-5p | 1.0969 |
| hsa-miR-4651 | 1.2055 |
| hsa-miR-4533 | 0.9166 |
| hsa-miR-4475 | 0.8424 |

**Table S2.** List of differentially expressed cellular miRNAs in miR-K12-3-3p transfected HOK. Fold change values compared with control mimic are provided. MiRNAs with ‘°’ denotes FDR (Benjamini Hochberg Test) adjusted significance of p<0.05.

| **Annotation** | **Fold Change** |
| --- | --- |
| hsa-miR-1226-3p | 1.8264 |
| hsa-miR-124-3p | 2.0873 |
| hsa-miR-1255a | 1.3943 |
| hsa-miR-148b-3p | 1.4037 |
| hsa-miR-23a-5p | 9.6272 |
| hsa-miR-27a-5p | 1.0941 |
| hsa-miR-3935 | 2.1568 |
| hsa-miR-4258 | 1.2766 |
| hsa-miR-4652-3p | 3.3023 |
| hsa-miR-4725-5p | 0.2772 |
| hsa-miR-513b | 1.5599 |
| hsa-miR-5704 | 1.4047 |
| hsa-miR-936 | 2.4697 |

**Table S3.** List of differentially expressed cellular miRNAs in miR-UL70-3p transfected HOK. Fold change values compared with control mimic are provided.

| **Annotation** | **Fold change** |
| --- | --- |
| hsa-miR-4436b-3p° | 70.7780 |
| hsa-miR-4450° | 164.6993 |
| hsa-miR-5006-3p° | 265.1855 |
| hsa-miR-647° | 34.3919 |
| hsa-miR-4426° | 76.6648 |
| hsa-miR-4706° | 16.3575 |
| hsa-miR-4669° | 23.7107 |
| hsa-miR-4795-5p° | 18.9502 |
| hsa-miR-4657° | 49.1716 |
| hsa-miR-4764-5p° | 4.3679 |
| hsa-miR-4477b° | 6.4541 |
| hsa-miR-5004-5p° | 13.2878 |
| hsa-miR-4314° | 4.4514 |
| hsa-miR-4275° | 2.3755 |
| hsa-miR-3156-3p° | 2.5747 |
| hsa-miR-518e-5p/hsa-miR-519a-5p/hsa-miR-519b-5p/hsa-miR-519c-5p/hsa-miR-522-5p/hsa-miR-523-5p° | 2.4709 |
| hsa-miR-1255b-2-3p° | 2.1900 |
| hsa-miR-7-5p° | 2.6983 |
| hsa-miR-483-5p° | 1.9048 |
| hsa-miR-630° | 1.7836 |
| hsa-miR-429* | 1.3344 |
| hsa-miR-21-3p° | 1.4579 |
| hsa-miR-5584-3p° | 1.4626 |
| hsa-miR-4725-5p | 0.1364 |
| hsa-miR-4756-3p | 1.2482 |
| hsa-let-7f-1-3p | 1.1994 |
| hsa-miR-1260a | 1.1835 |
| hsa-miR-205-3p | 1.2297 |
| hsa-miR-4501 | 0.8640 |
| hsa-miR-423-3p | 0.8624 |
| hsa-miR-492 | 0.8665 |
| hsa-miR-1255a | 0.7358 |
| hsa-miR-4288 | 0.8030 |
| hsa-miR-4511 | 0.5743 |
| hsa-miR-3911 | 0.8804 |
| hsa-miR-3924 | 0.8752 |
| hsa-miR-9-5p | 1.1269 |
| hsa-miR-4478 | 0.8356 |
| hsa-miR-4297 | 0.8817 |
| hsa-miR-4505 | 0.7751 |
| hsa-miR-552 | 0.7283 |
| hsa-miR-30e-3p | 1.1162 |
| hsa-miR-495-5p | 0.8551 |
| hsa-miR-210 | 0.8744 |
| hsa-miR-302a-3p | 0.7941 |
| hsa-miR-4475 | 0.8114 |
| hsa-miR-4540 | 0.8802 |
| hsa-miR-4778-5p | 0.8758 |
| hsa-miR-490-3p | 0.8329 |
| hsa-miR-516b-5p | 0.8991 |
| hsa-miR-31-3p | 1.1400 |
| hsa-miR-1973 | 0.8915 |
| hsa-miR-203a | 0.8584 |
| hsa-miR-27a-5p | 1.1264 |
| hsa-miR-3124-3p | 0.7742 |
| hsa-miR-3136-3p | 0.8084 |
| hsa-miR-3676-5p | 1.0963 |
| hsa-miR-3915 | 0.8641 |
| hsa-miR-4299 | 0.8188 |
| hsa-miR-4636 | 0.6641 |
| hsa-miR-4644 | 0.8448 |
| hsa-miR-4716-5p | 1.1130 |
| hsa-miR-1226-3p | 0.8049 |

**Table S4.** List of differentially expressed cellular miRNAs in miR-H1 transfected Mφ. Fold change values compared with control mimic are provided. MiRNAs with ‘°’ denotes FDR (Benjamini Hochberg Test) adjusted significance of p<0.05.

| **Annotation** | **Fold Change** |
| --- | --- |
| hsa-miR-489° | 31.7484 |
| hsa-miR-1184° | 13.7087 |
| hsa-miR-4659b-3p° | 3.5354 |
| hsa-miR-943° | 6.6020 |
| hsa-miR-4451° | 1.6326 |
| hsa-miR-21-3p° | 1.6370 |
| hsa-miR-23a-3p° | 0.4340 |
| hsa-miR-4288° | 0.3981 |
| hsa-miR-155-5p° | 1.4501 |
| hsa-miR-4497° | 1.5623 |
| hsa-miR-26a-2-3p° | 1.2867 |
| hsa-miR-155-3p | 2.5015 |
| hsa-miR-3178 | 1.4655 |
| hsa-miR-107 | 1.2983 |
| hsa-miR-30a-5p | 1.3505 |
| hsa-miR-425-5p | 0.8398 |
| hsa-miR-423-3p | 0.8026 |
| hsa-miR-513a-3p | 3.1332 |
| hsa-miR-26a-5p | 1.3693 |
| hsa-let-7a-5p | 1.3146 |
| hsa-miR-1260b | 0.8201 |
| hsa-miR-4289 | 1.4706 |
| hsa-miR-3685 | 0.7248 |
| hsa-miR-423-5p | 1.2112 |
| hsa-miR-664a-3p | 0.8611 |
| hsa-miR-1249 | 0.8548 |
| hsa-miR-23a-5p | 0.8629 |
| hsa-miR-3142 | 1.1535 |
| hsa-miR-361-3p | 1.2334 |
| hsa-miR-4317 | 1.2291 |
| hsa-miR-29a-3p | 1.4516 |
| hsa-miR-195-5p | 1.2757 |
| hsa-miR-4454 | 0.8658 |
| hsa-miR-936 | 0.8867 |
| hsa-miR-874 | 1.3539 |
| hsa-miR-1469 | 1.1577 |
| hsa-miR-191-5p | 1.2034 |
| hsa-miR-326 | 0.8443 |
| hsa-miR-378b | 1.2707 |
| hsa-miR-4456 | 0.7091 |
| hsa-miR-4657 | 0.6089 |
| hsa-miR-513b | 0.8311 |
| hsa-miR-4436b-5p | 0.8638 |
| hsa-miR-335-3p | 0.8189 |
| hsa-miR-186-5p | 1.1850 |

**Table S5.** List of differentially expressed cellular miRNAs in miR-K12-3-3p transfected Mφ. Fold change values compared with control mimic are provided. MiRNAs with ‘°’ denotes FDR (Benjamini Hochberg Test) adjusted significance of p<0.05.

| **Annotation** | **Fold Change** |
| --- | --- |
| hsa-miR-23a-5p° | 18.8187 |
| hsa-miR-936° | 3.6886 |
| hsa-miR-3935° | 2.4739 |
| hsa-miR-513b° | 1.8492 |
| hsa-miR-1255a° | 1.5473 |
| hsa-miR-4258 | 1.7245 |
| hsa-miR-4508 | 1.6658 |
| hsa-miR-1260a | 1.3814 |
| hsa-miR-1587 | 1.1666 |
| hsa-miR-7-5p | 0.7764 |
| hsa-miR-4530 | 1.3242 |
| hsa-miR-1264 | 1.2225 |
| hsa-miR-142-3p | 0.7300 |
| hsa-miR-148b-3p | 1.1921 |
| hsa-miR-15b-5p | 0.8213 |
| hsa-miR-17-5p | 0.7425 |
| hsa-miR-181d | 0.8009 |
| hsa-miR-1913 | 0.8560 |
| hsa-miR-19a-3p | 0.8487 |
| hsa-miR-21-3p | 1.2010 |
| hsa-miR-33a-5p | 0.7971 |
| hsa-miR-3675-3p | 0.8375 |
| hsa-miR-371b-5p | 1.4553 |
| hsa-miR-3940-5p | 1.4158 |
| hsa-miR-3960 | 1.6107 |
| hsa-miR-425-5p | 0.8688 |
| hsa-miR-4279 | 1.5124 |
| hsa-miR-4285 | 1.4410 |
| hsa-miR-4286 | 1.2675 |
| hsa-miR-4468 | 1.1506 |
| hsa-miR-4484 | 1.3598 |
| hsa-miR-4497 | 1.2262 |
| hsa-miR-4516 | 1.3120 |
| hsa-miR-4644 | 1.2249 |
| hsa-miR-4708-3p | 1.5244 |
| hsa-miR-4787-5p | 1.5874 |
| hsa-miR-4800-3p | 1.5308 |
| hsa-miR-5704 | 1.1868 |
| hsa-miR-665 | 1.2519 |
| hsa-miR-718 | 0.8387 |
| hsa-miR-98-5p | 0.7469 |

**Table S6.** List of differentially expressed cellular miRNAs in miR-UL70-3p transfected Mφ. Fold change values compared with control mimic are provided. MiRNAs with ‘°’ denotes FDR (Benjamini Hochberg Test) adjusted significance of p<0.05.
